# Stigma and discrimination faced by adolescents living with HIV and experiencing depression in Malawi

**DOI:** 10.1186/s44263-024-00072-3

**Published:** 2024-07-01

**Authors:** Maria Faidas, Melissa A. Stockton, Steven M. Mphonda, Griffin Sansbury, Haley Hedrick, Jackson Devadas, Twambilile Phanga, Laura Ruegsegger, Jack Kramer, Hillary Mortensen, Kazione Kulisewa, Brian W. Pence, Nivedita L. Bhushan, Bradley N. Gaynes

**Affiliations:** 1 University of North Carolina at Chapel Hill, 321 S Columbia St., Chapel Hill, NC 27599 USA; 2 University of Pennsylvania, Philadelphia, PA USA; 3 University of North Carolina Project Malawi, Lilongwe, Malawi; 4 University of Miami, Miami, FL USA; 5 University of North Carolina at Greensboro, Greensboro, NC USA; 6 Kamuzu University of Health Sciences, Blantyre, Malawi; 7 Research Triangle Institute International, Research Triangle Park, NC USA

**Keywords:** Stigma, Discrimination, Adolescents, HIV, Depression, Malawi

## Abstract

**Background:**

In Malawi, approximately 25% of adolescents living with HIV (ALWH) also suffer from depression. Not only is HIV stigma a major contributor to depression but it also adversely impacts HIV care engagement. ALWH can experience HIV stigma as stereotyping, social exclusion, low social support, and abuse, and these experiences are associated with poor mental health. Despite recognition of the deleterious effects of HIV stigma, we have limited knowledge of how stigma is experienced by ALWH with comorbid depression. Guided by the Health Stigma and Discrimination Framework, we describe stigma faced by ALWH and comorbid depression in Malawi and its implications for future interventions.

**Methods:**

As part of a larger formative study to adapt a mental health counseling intervention, we conducted in-depth interviews, social support mapping sessions, and focus-group discussions with 25 ALWH, 4 caregivers of ALWH, 3 HIV providers, and 5 participants and 5 staff of a prior mental health counseling intervention. After analyzing the stigma codes, we used the Health Stigma and Discrimination Framework to organize the data into four key domains: drivers, manifestations, outcomes, and health and social impacts.

**Results:**

Major drivers of HIV stigma included fear of HIV transmission, negative effects of antiretroviral therapy (ART), association with death, inaccurate knowledge, and negative attitudes towards ALWH. The most common manifestations of HIV stigma were gossip, insults and mocking, and physical and social distancing. Decreased ART adherence and missed HIV appointments were commonly cited outcomes of HIV stigma. Broader health impacts of HIV stigma were notable for mental health comorbidities including depression, anxiety, substance use, and suicidality. Identified resilience strategies included support for HIV care engagement and psychosocial support from family and friends.

**Conclusions:**

This study systematically describes the stigmatization process faced by ALWH and experiencing depressive symptoms in Malawi. Notably, HIV stigma continues to disrupt HIV care and detrimentally impacts mental health during adolescent development. Further studies focused specifically on stigma are needed to better characterize this process and identify additional resilience factors. Investment in stigma-reduction interventions for ALWH is needed to avert poor mental health and HIV outcomes.

**Supplementary Information:**

The online version contains supplementary material available at 10.1186/s44263-024-00072-3.

## Background

HIV remains the leading cause of death amongst adolescents living in much of Southern Africa [1]. Despite widely available effective HIV treatment, only 65% of adolescents living with HIV (ALWH) globally are on antiretroviral therapy (ART), and they have much lower rates of ART adherence and viral suppression compared to adults and children [2, 3]. These low rates may reflect the unique challenges faced by ALWH. ALWH must navigate complex health care requirements and social dynamics inherent to living with HIV, all while undergoing a critical and accelerated period of psychological, social, and physical development [4]. Poor mental health, such as depression, and stigma, also adversely impact on ART adherence [5–7].

Stigma is a complex social process that leads to the social and economic exclusion of individuals or groups based on real or perceived differences [8, 9]. Discrimination has been conceptualized as the endpoint of the stigmatization process, referring to the unjust and unfair treatment of an individual based on these perceived differences [10, 11]. Stigma is particularly harmful in the context of living with HIV [5, 12–15], as it can adversely impact appointment attendance and ART adherence [13–17], and often exacerbates poor mental health [18]. HIV stigma is particularly important to understand and address during adolescence as ALWH are in a critical period of development and change [4, 19].

Recent studies have suggested elevated depressive symptoms amongst people living with HIV. While the use of symptom severity scales may inflate depression prevalence [20], estimates of comorbid depression and HIV in sub-Saharan Africa (SSA) range from 8 to 15% [21, 22]. This is particularly the case in Malawi, where the prevalence of depression amongst ALWH is high (18–26%) [23], and may be due to multitude of factors, namely living with a chronic, highly stigmatized disease. However, there is limited evidence in this context of how ALWH experience HIV stigma. In the only study in Malawi to purposely examine stigma in ALWH, participants reported stereotyping, discrimination, social exclusion, and abuse, all of which contribute to depression [16]. Fear of status disclosure and psychological abuse contributed to poor ART adherence [16]. This study further identified stigma as an overlooked barrier to HIV care, calling for future research efforts in this area. Because stigma is culturally and contextually distinct [12], there is a need to better understand how stigma manifests and is perpetuated in the Malawian context before developing relevant interventions for ALWH.

The Health Stigma and Discrimination (HSD) Framework is a global, crosscutting theoretical framework used to systematically describe the stigmatization process across various socioecological levels [24]. It has been used to guide intervention development, measurement, research, and policy efforts, and has been applied to a range of health conditions, including both HIV and mental illnesss [24]. Further, the framework is particularly relevant in this context as it can be used to describe the entire stigmatization process, from causes of stigma (drivers), stigma itself (manifestations), the immediate impact of stigma (outcomes), and ultimately the larger effect stigma has on health and well-being (health & social impacts). In the field of stigma research, elucidating the entire stigmatization process is necessary to build context specific programs that intervene at the most salient parts of the stigmatization process to decrease stigma and ultimately improve health outcomes.

In this study, we use qualitative data and draw on the HSD Framework to explore the stigmatization process faced by ALWH with comorbid depression in Malawi. We describe the drivers, manifestations, outcomes, and health and social impacts of HIV-related stigma and then discuss the implications of these findings for future interventions to reduce stigma amongst this population.

## Methods

### Study context and design

This manuscript presents analysis of qualitative data collected during the formative phase of HEADS-UP [25], a study focused on adapting a counseling and peer support intervention for ALWH experiencing depressive symptoms in Lilongwe, Malawi. Specifically, the formative study gathered data to adapt and augment the Friendship Bench (FB) [26, 27], an evidence-based, problem-solving counseling intervention for common mental disorders that has previously been tailored for the Malawian setting [28–32]. The formative phase of HEADS-UP serves as the groundwork to inform a subsequent pilot trial of the adapted FB intervention. The formative study was conducted in three government health care facilities in Lilongwe, Malawi, with similar staffing levels, services offered, NGO involvement, and ALWH patient volume. Each of the three clinics also provided an adolescent-specific ART program called “Teen Clubs”. Teen Clubs meet one weekend day every 3 months to provide ART refills, viral load testing, and psychosocial support to ALWH between the ages of 10–19. Prior to initial Teen Club enrollment, all ALWH are educated on HIV and made aware of their serostatus. The ongoing psychosocial support includes education on ART adherence, nutrition, and sexual and reproductive health, and youth-friendly activities. All clinics provided age differentiated Teen Clubs for patients aged 10–14 and patients aged 15–19.

### Study sample and recruitment

All participants were selected using purposive sampling. ALWH were eligible to participate if they were as follows: (1) age 13–19, (2) living with HIV, and (3) screened positive for depressive symptoms with a score ≥ 13 on the Beck Depression Inventory-II (BDI-II). All ALWH aged 13–19 present at the Teen Clubs were screened for depressive symptoms with the BDI-II administered by either clinical staff or the research assistants. Amongst all ALWH screened, 24.7% met the criteria for depressive symptoms. The BDI-II is a 21-item screening tool for assessing common depressive symptoms such as low mood, anhedonia, disturbed sleep, and changes in appetite [33]. BDI-II scores classify reported depressive symptoms as minimal (0–13), mild (14–19), moderate (20–28), and severe (29–63) [33]. The BDI-II was validated for ALWH in Malawi, with a cutoff ≥ 13 demonstrating 80% sensitivity in detecting depressive symptoms [23]. Interviews were conducted the same day if a guardian was present to provide consent or scheduled within 2 weeks (e.g., the recall period for the BDI-II) to allow for a guardian to provide consent. A biological parent or other identified primary caregiver was considered a guardian. AWLH were asked to provide their guardian’s phone number, if known, or research assistants provided a call-back number. Research assistants explained the study to guardians via phone, and if interested, guardians were asked to present in-person to the health facility with their ALWH for written consenting. Research assistants contacted ALWH to schedule FGDs for a later date.

All ALWH who screened positive for depression, whether or not they chose to participate, were referred same day to a psychosocial counselor on-site. Any ALWH who disclosed suicidal ideation was referred same day to a research assistant for immediate assessment to determine suicide risk and additionally to a psychosocial counselor for further work-up and safety planning.

In addition to ALWH, the formative study also included caregivers, HIV providers, and young adult participants (aged 18–25) and staff of Periscope, a prior FB intervention for perinatal women living with HIV and comorbid depression [28]. Both caregivers and providers were included as key stakeholders in this study as they provide social and health-related support for ALWH. Caregivers were recruited for a focus-group discussion during the consenting process for their ALWH participant. Providers were recruited by phone for a separate focus group discussion directly from the clinics. Periscope staff (interventionists and research staff) and participants (women living with HIV, age 18–25, previously diagnosed with perinatal depression who received FB counseling) were also included this study. They were regarded as key stakeholders, given their prior involvement in facilitating the FB (for staff), or proximity in age to the ALWH, and experience engaging in a similar FB intervention. The research coordinator recruited Periscope participants and staff by phone. Of note, there were no instances of participant study refusal or dropout.

### Data collection

We conducted 20 in-depth interviews (IDIs), 10 social support network mapping sesssions, and 3 focus-group discussions (FGDs) between January and April 2023 (Table 1). For the IDIs and FGDs, four semi-structured interview guides were developed to explore experiences with depression, HIV care, social support, HIV-related stigma, and preferences for various peer support strategies to support HIV care engagement (Additional file 1: Interview Guides; Additional file 1: Table S1). For the social support mapping sessions, a study team member guided the participant through a 60-min exercise and discussion that elicits the structure and function of their personal social support network, with specific probes for how individuals in their network impacted their HIV care and mental health (Additional file 1: Interview Guides) [34–36]. After piloting the interview guides and reviewing the transcripts, only small wording changes were made to the guides for easier comprehension. The IDIs, social support mapping sessions, and FGD lasted between 1 and 1.5 h and were all conducted by research assistants in Chichewa, the national language of Malawi, in a private location at the clinic study sites. Although some participants can speak English, we opted to conduct all interviews in Chichewa for ease of self-expression in participants’ native language. All three data collection activities were digitally recorded, transcribed in Chichewa, and then translated into English by study staff according to a transcription protocol. All transcripts were reviewed by the interviewer for transcription and translation accuracy.

**Table 1:** Data collection activities

**Participant type**	**Data collection activities**
**ALWH**^**a**^** (*n* = 25)**	10 in-depth interviews 10 social support mapping sessions 1 focus-group discussion
**Stakeholders (*n* = 7)**	
Caregivers (*n* = 4)	1 focus-group discussion
Providers (*n* = 3)	1 focus-group discussion
**Participants and staff in the Periscope intervention (*n* = 10)**	
Participants (*n* = 5)	5 in-depth interviews
Staff (*n* = 5)	5 in-depth interviews

^a^Adolescents living with HIV

### Analysis

Data processing, coding, and analysis involved four steps: (1) reading for content, (2) deductive and inductive coding, (3) data display to identify emerging themes, and (4) interpretation [37, 38]. The English translated transcripts were used for data analysis. After reading each of the transcripts, two study team members collaboratively drafted a thematic codebook that deductively captured important topics covered in the data collection guides and inductively captured emerging themes. This initial codebook was reviewed and refined by the entire research team. Codes related to HIV stigma included fear of HIV status disclosure, mocking, social isolation, and differential and unfair treatment.

All coding was conducted after the completion of interviews. Coding was an interative process in which the codebook was revised based on coder’s feedback and weekly team meetings. Six study members each applied the initial codebook to two transcripts using Dedoose [39] and then met to conduct a line-by-line comparison of code application, discuss challenges, and refine the codebook. After revising the codebook, the same team members repeated the process two more times, at which point inter-rater reliability was deemed acceptable. The final codebook was used to double code the remaining transcripts by five of the study team members (Additional file 2). After coding was complete, a team member generated a brief memo describing themes related to stigma. Two study team members then reviewed all codes related to stigma from ALWH, caregivers of ALWH, HIV providers, and Periscope participants and staff.

Two study team members conducted thematic analysis [40] to describe the stigmatization process using the HSD Framework as a guide to structure the analysis and presentation of the results [24]. Broadly, the HSD Framework conceptualizes the stigmatization process in four constituent domains: (1) drivers, (2) manifestations, (3) outcomes, and (4) health and social impacts. Drivers are factors that drive health-related stigma, such as fear, lack of awareness, stereotypes, and prejudice. Notably, stereotypes and prejudice can also be present as manifestations, however are treated as drivers in this analysis. Manifestations refer to stigma and discrimination that occur after a label is applied to a person with an undesirable difference. There are different types of stigma which manifest in distinct ways [9]. *Experienced stigma* refers to one’s personal experience with stigmatizing behaviors, whereas *witnessed stigma* describes the viewer perspective of stigma being perpetrated against someone else. *Anticipated stigma* is the expectation of bias if one’s health condition becomes known. *Internalized stigma*, or self-stigma, is the adoption of negative societal beliefs by the stigmatized individual and is often one of the downstream manifestations after repeated instances of experienced or witnessed stigma. Outcomes include various consequences for affected populations, including adherence to treatment, resilience, and advocacy. Health and social impacts include individual- and population-level implications of health-related stigma, including morbidity and mortality.

Of note, all qualitative methods align with the COREQ (COnsolidated criteria for REporting Qualitative research) checklist (Additional file 3) [41].

## Results

### Participant characteristics

There were 42 total participants in this study. The 25 ALWH had a near equal distribution by gender (*n* = 12 females and *n* = 13 males). The mean age of ALWH was 16.2 years, with 64% (*n* = 16) οf participants in the 16–19 age group. With respect to education, 32% (*n* = 8) of participants had completed secondary school, with the remaining having only completed primary school. Only 40% (*n* = 10) of participants had two living biological parents. Nearly 75% (*n* = 18) of participants reported food insecurity, and 64% (*n* = 16) reported money insecurity in the past year. Participant BDI-II scores ranged from 13 to 50 (maximum 63). The mean BDI-II score was 19.4, near the moderate severity cutoff of 20. Females had a slightly higher BDI-II score at 21.4, compared to 18.0 in males. The majority of participants (76%, *n* = 19) self reported mother-to-child transmission (MTCT) of HIV, with the remaining unsure of how they acquired HIV (Table 2).

**Table 2:** Characteristics of the ALWH participants

*n* (%)	Total ALWH (*n* = 25)		Female ALWH (*n* = 12)	Male ALWH (*n* = 13)
Age (mean)		16.2	16.4	15.9
13–15		9 (36)	3 (25)	6 (46.2)
16–19		16 (64)	9 (75)	7 (53.8)
Education				
Primary		17 (68)	6 (50)	11 (84.6)
Secondary		8 (32)	6 (50)	2 (15.4)
Both parents alive		10 (40)	6 (50)	4 (30.1)
Past year food insecurity		18 (72)	7 (58.3)	11 (84.6)
Past year money insecurity		16 (64)	6 (50)	10 (76.9)
BDI-II score (mean)		19.6	21.4	18.0
Self-reported HIV transmission				
MTCT^a^		19 (76)	9 (75)	10 (76.9)
Do not know		6 (24)	3 (25)	3 (25)

^a^Mother to child transmission

The mean age of caregivers was 47.8 years, with an even gender divide. More than half of caregivers were married, and their professions included gardener, landscaper, teacher, and businesswoman. HIV providers included nurses and clinicians with a mean age of 38.3 years. All Periscope participants were female, and the mean age was 23.2 years. Periscope staff included four psychosocial counselors and one study coordinator with a mean age of 34 years.

### Stigma and discrimination faced by ALWH and comorbid depression in Malawi

**Fig. 1:**
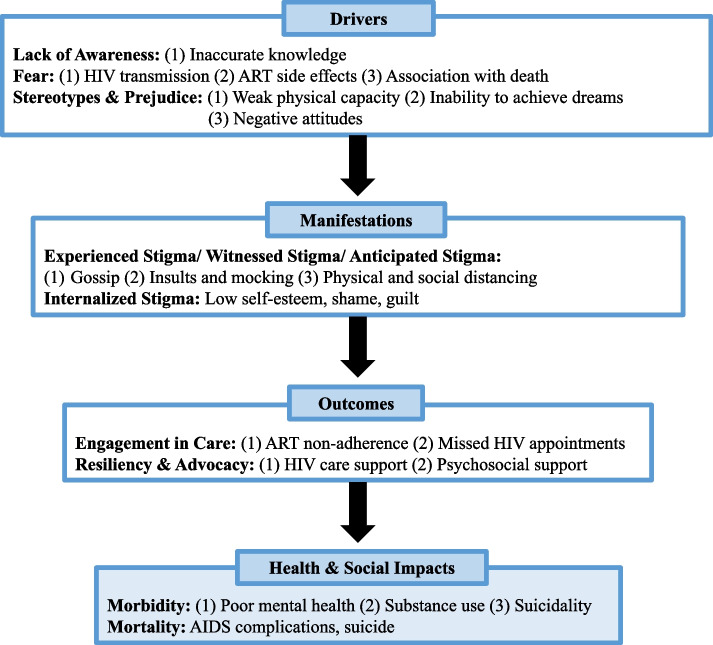
Health Stigma and Discrimination Framework, adapted for ALWH and comorbid depression in Malawi

We structured our results around each of the four key domains of the HSD Framework: drivers, manifestations, outcomes, and health and social impacts of stigma amongst ALWH (Fig. 1). Within the drivers domain, fear, lack of awareness, and stereotypes and prejudice emerged as sub-domains. Manifestations include experienced, witnessed, anticipated, and internalized stigma. Within outcomes, sub-domains include engagement in care and resilience and advocacy. Within health and social impacts, sub-domains include morbidity and mortality. In the following sections, we describe themes relevant to each sub-domain.

### Drivers

#### Lack of awareness

##### Inaccurate knowledge

The key driver underlying HIV stigma was inaccurate knowledge about modes of HIV transmission, the harmful nature of ARTs, and HIV prognosis. This participant describes how inaccurate knowledge may drive stigmatizing behaviors.

*“Some may choose a cup and give him food separately not to mix with the others…for fear of being infected with the disease he has.”* – Periscope participant, female, age 25

In this example, stigmatizing behaviors are driven by the fear that HIV can be transmitted through sharing cups and utensils. As discussed in the following sections, widespread inaccurate knowledge underpins much of the fear around HIV transmission, ARTs, and death.

#### Fear

##### Fear of HIV transmission

The public’s fear of HIV transmission was the most commonly reported driver of HIV stigma and was reported by both male and female ALWH and Periscope participants. Participants stated that this fear of HIV transmission often results in others avoiding physical touch with individuals living with HIV. Many participants remarked that sores and skin rashes are of particular concern, as it is a visible sign of HIV that further intensifies the fear of HIV transmission.

*“What I fear is if I don’t take my medication, I might develop sores, or rashes on my skin… I am afraid that it can be difficult for me to mix with my friends and come near them… they think you will infect them.”* – ALWH, male, age 15, BDI-II 19 (moderate)

These distancing measures taken by people without HIV are further reflective of the pervasive inaccurate knowledge about modes of HIV transmission.

##### Fear of ART side effects

Participants from all stakeholders groups, except HIV providers, spoke to the negative attitudes around ARTs. Participants spoke to the potentially harmful side effects of ARTs, a fear that is held notably by both people living with and without HIV.

*“They have a misconception that maybe if I continue taking this medication, I will die a sudden death, and because of this they choose not to take their medication.”* – ALWH, male, age 17, BDI-II 18 (mild)

*“Others are afraid of having the drugs accumulating in their stomach, causing damage, and this being the main cause of sudden death… as such, some stop taking their medication.”* – Periscope participant, female, age 25

Despite recognition that the harmful nature of ART medications is a misconception, the public’s opinion of ARTs creates fear amongst ALWH that these medications are abnormal and unsafe. This fear of ARTs further contributes to negative attitudes towards engaging in HIV care and taking ARTs.

##### Fear/association with death

Many participants spoke to the innacurate public notion that having HIV is a death sentence. This fear is perpetuated in school, home, and community settings. In schools, students are taught from a young age that HIV kills.

*“Schools take HIV as a killer... the child only knows that HIV kills. The school syllabus doesn’t say anything that a person can be positive and still have a future and have children. So things like these are contributing to our youths having concerns. At school, I was taught this and when I come to the clinic, they are telling me this… Like we used to sing, “AIDS! AIDS! AIDS! Is a killer!”* – HIV clinician, female, age 38

This quote exemplifies the societal underpinning that “HIV is a killer”. This HIV clinician references the fact that schools continue to teach that HIV is a fatal disease, over 20 years since this participant was in grade school. HIV’s association with death further drives the fear of HIV transmission.

#### Stereotypes and prejudice

##### Weak physical capacity

Male ALWH spoke about the commonly held belief that people living with HIV are physically weak, unhealthy, and have a shortened lifespan. Notably, this stereotype was not raised by female ALWH. This male ALWH describes how this stereotype manifests in community settings where men are expected to participate in manual labor.

*“A youth who has HIV cannot manage to push his or her dream forward and cannot do a tiresome task, like digging graves. For instance, if someone says, “Take this one to help dig,” others will say, “Do you want your friend to continue getting sick?” People talk like that… When you want to help dig by removing dirt in the grave hole, some say, “This one can’t manage, his body is already finished. Do you want us to bury two people here?” That’s how it goes.”* – ALWH, male, age 18, BDI-II 21 (moderate)

This quote exemplifies the negative stereotypes that are held about males living with HIV; that they are weak and illness prone, and thus incapable of participating in physical labor and fulfilling their societal expectations.

##### Inability to achieve dreams

The stereotype that a person living with HIV is unable to achieve their dreams follows from the stereotype that they are physically weak and have a shortened lifespan. In the quote that follows, a caregiver of an ALWH shares a story of his mother telling him that he will not live long enough to eat fruits from a tree he planted.

*“Before I started taking my ARTs, I planted a Masuku tree, and my mum said to me, “You are planting that tree. Will you even have a chance to eat its fruits. Who will eat them for you?” I said to her, “Mum, I shall eat them, and for your information, you shall die while I still live.” Of course, she died and I am eating Masuku.”* – caregiver of ALWH, male, age 31

This metaphor speaks to the presumed poor prognosis of a person living with HIV. Planting a tree can represent goal setting and future-oriented tasks. Eating fruits from a tree one has planted can symbolize achieving one’s dreams. In this example, the mother implies that he will not live long enough to eat the fruits and achieve his dreams. Notably, this view is held by a mother of a former ALWH, which exemplifies how a stereotype is carried forward throughout many generations.

##### Negative attitudes

Many participants discussed the pervasive negative attitudes towards ALWH, for no reason other their HIV status. Even without knowing someone’s character, a community member may judge and think negatively about ALWH due to their HIV status. A caregiver explains how community members are prejudiced towards an ALWH:

*“I see that discrimination is common in our communities, and this makes people feel unwanted and less important. This comes when people discover that you are HIV positive and you are on ART; they develop a certain hate towards you and they don’t want you near them, so they discriminate you.”* – caregiver of ALWH, female, age 47

These negative attitudes are pervasive in community settings, often without cause, but may also be a direct result of the previously discussed drivers, including fear, lack of awareness, and negative stereotypes.

### Manifestations

#### Experienced stigma/Witnessed stigma/Anticipated stigma

This section describes experienced, witnessed, and anticipated stigma. When discussing manifestations of stigma, only a few participants spoke directly about personal experiences of stigma. Instead, most participants spoke about witnessed stigma, which in turn resulted in anticipated stigma.

##### Gossip

The most common manifestation of HIV stigma discussed was gossip. Nearly all participants, including ALWH from both sexes and age groups, reported gossip as the most feared manifestation following HIV status disclosure. This female ALWH describes how gossip can spread in the community.

“However, when you part ways with the person, he or she can go to his or her friends and start saying that Thandi* takes ARTs. “I escorted her today (to the clinic), I know that she receives (ARTs).” The news that Thandi takes ARTs can now spread.” – ALWH, female, age 17, BDI-II 16 (mild) *name has been changed

An individual’s HIV status is regarded as highly private information and a noteworthy piece of gossip. The ensuing gossip after HIV status disclosure can be devastating to an ALWH. As discussed in the “Outcomes” section, ALWH do not want to be seen at a health clinic, as community members may make assumptions about their HIV status and spread the news through the community.

##### Insults and mocking

Participants commonly reported insults and mocking from both friends and family. The insults could be provoked or unprovoked, as illustrated in the quotes below. This male ALWH explains that after a fight or misunderstanding with his parents, his parents capitalize on this opportunity to insult him:

*“Many people, my friends, and even my parents get so comfortable and say that I am good for nothing… mock me comparing me to my siblings and saying they are far better than me and they are obedient.”* – ALWH, male, age 19, BDI-II 17 (mild)

In the prior example, his parents compared him to his siblings (who do not have HIV) and attribute his “bad behavior” to his HIV status. In the example that follows, another participant notes that his peers mock him for taking ART medications, without a justified reason, and for the sole intent of causing distress.

*“Some know your secret, that you are in this situation— so they just say something to disturb you. They can say something concerning your (HIV) status like, “This (person) takes medication every day, what kind of person is that.” You see, things like that.”* – ALWH, male, age 17, BDI-II 13 (minimal)

Use of ART medications is employed as grounds for insulting and imparting shame, due to the negative association of ARTs with HIV.

##### Physical and social distancing

Participants also commonly reported physical distancing, with specific mentions of refusing to eat, bathe, and socialize with ALWH, all driven by the fear of HIV transmission. This physical distancing was reported in home, school, and community settings. A Periscope participant shares an example of distancing within the family unit:

*“They say a lot of words like “You can’t be my brother, especially one who is on ARTs.” For example, it happened to me that my own sister said, “Don’t come near me, you have HIV. There should be social distance between us.” … I got counseling.”* – Periscope participant, female, age 21

In this example, a person living with HIV explains how her sibling without HIV refused to socialize with her within the home. This experience was so distressing that she received counseling. Participants also noted that these stigmatizing behaviors are also often enacted by a stepparent towards an ALWH, which often becomes the grounds for abuse and neglect.

#### Internalized stigma

Internalized stigma, or self-stigma, primarily manifested as low self-esteem, shame, and guilt. This form of stigma was reported by all participants, including ALWH, caregivers, and Periscope participants. In this process, an individual begins to identify with the negative stereotypes and treatment of individuals living with HIV, thus adversely influencing their self-esteem. As described by one male ALWH as follows:

*“They say that those who take ARTs should not chat with us and we are playing only with the ones who don’t take ARTs… so this makes you think a lot and ask questions like “Why did this come to me?” or “Who are you?” which make you feel guilty.”* – ALWH, male, age 17, BDI-II 18 (mild)

This participant notes that his peer’s refusal to chat and socialize with him produces a sense of shame and guilt, which contributes to the development of internalized stigma. In the example below, another male ALWH describes a similar process and directly cites stigma marking via HIV status as a contributor to internalized stigma.

*“Maybe sometimes when someone says I am worthless it gives me a lot of thoughts that maybe is it because of my status… You start attributing it to some things that differentiates us, like [HIV] status …. So, I ask myself questions …. with no answer you start feeling worthless… that ahhhh maybe it would have been better if I wasn’t born here… so it is tough.”* – ALWH, male, age 19, BDI-II 17 (mild)

This ALWH describes his personal process of facing stigma from others, accepting those critiques, and how it impacts the way he perceives and values himself. Notably, an individual may keep their HIV status a secret, but still experience internalized stigma, as it is a deeply personal process and often driven by witnessed stigma.

### Outcomes

#### Engagement in care

##### ART nonadherence

Many participants report that HIV stigma is a major contributing factor to ART nonadherence. In particular, ARTs are heavily stigmatized primarily because being seen taking ARTs reveals one’s HIV status but also due to the belief that they are dangerous medications that can cause a sudden death. In the following example, a male ALWH describes how stigma directly impacts his nonadherence to ARTs.

*“When you are mocked by friends and they say “Go away, you take ARTs,” that makes you think too much like, “I take ARTs, these are not normal drugs”… As a confidence booster, you can say, “I don’t take ARTs”…*

*They spread that this one has HIV and convince everyone that they should not associate with you and they call you different names…. It is so discouraging and you end up getting frustrated and consider not to continue taking your drugs [ARTs]… you end up just stopping to take the medication.”* – ALWH, male, age 17, BDI-II 18 (mild)

This ALWH reflects that community members perceive ARTs as abnormal drugs. In a healthy adolescent with suppressed viral load, ARTs serve as a visible marker of an illness that may otherwise be invisible. ART use denotes a “diseased” state, so identifying ART use in the stigmatization process functions to mark ALWH as “different.” Thus, an ALWH may choose to stop taking their ARTs in an effort to hide their HIV status, fit in with their peers, and avoid further stigmatization. In the quote that follows, an HIV provider explains how depression driven by stigma can also contribute to ART nonadherence.

*“Mainly it’s because of feeling sorry for oneself that I’m taking medication. Some, it’s because they lose heart that they will be taking medication for the rest of their lives. So, they say it’s better to just stop taking medication and die. They see themselves as no longer important. They think they will be laughed at or they will be asked why they are taking medication every day. These make one to be depressed and stop taking medication.”* – HIV provider, male, age 42

Participants frequently reported discouragement around having to take a medication for the rest of their life. The responsibility of taking lifelong medications, compounded by the mocking around ART use, contributes to ART nonadherence. This HIV provider also references internalized stigma, in noting a lack of self-importance, as a factor in ART discontinuation. ALWH may choose death as a preferable option over a life overshadowed by stigma and societal rejection.

##### Missed HIV appointments

ALWH and Periscope participants also reported how HIV stigma can lead to decreased appointment attendance. As explained by one female ALWH as follows:

*“We can be shy to go to the place where medication is received [the clinic]… maybe when you go to receive medication, you can meet a friend.”*—ALWH, female, age 19, BDI-II 13 (minimal)

This ALWH explains that attending HIV appointments is a potential mechanism for inadvertent or involuntary HIV status disclosure. For this reason, she is hesitant to present to clinic to receive her ART medications. HIV appointment attendance can also be influenced by challenging family dynamics, where parents refuse to take their ALWH to the hospital or clinic. In this example, a caregiver explains how he was informed of such a scenario.

*“My daughter told me,“I have a friend and he doesn’t go to the hospital but is also HIV positive. His mum chased a [healthcare worker] who visits us and told her that she should mind her business. So the boy asked me, “Why does your mum support you and you have a good relationship? As for me, I have spent over 6 months without going to the hospital.” So my daughter asked him to come to me. The boy is afraid to come to me and I am also uncomfortable considering the negative reception he had towards the healthcare worker… this makes the boy sad since there is no support. Even if he stops taking his medication, there is no one to follow him and encourage him.”* – caregiver of ALWH, male, age 31

This example demonstrates a caregiver’s fear of being seen having a healthcare worker visit their home and later being identified as having a child living with HIV. This anticipated stigma creates further barriers to care for her child in accessing HIV care.

#### Resilience and advocacy

Social support, particularly from other ALWH, as well as from family and friends, was important for resiliency and negating the downstream adverse impacts of stigma. Identified support needs were understood as two distinct, yet interrelated ways: support for HIV care engagement and psychosocial support.

##### Support for HIV care engagement

ALWH reported that they often found support for HIV care engagement from other people living with HIV. One ALWH explains that her aunt, who is also living with HIV, supports her by providing advice on how to take ARTs, giving appointment reminders, and providing education on how to protect oneself from other communicable diseases. In this way, members of the same stigmatized group can encourage each other to remain engaged in their HIV care, despite opposing cultural norms.

*“The support is in advice form—advice about how to be taking ARTs, and also the way I can take care of myself. [She tells me] that I should be taking medication at the right time, and I should be going to the clinic to collect medication on the date they have given me… She says that there are other types of diseases that someone can infect you with apart from HIV. So, she tells me how I can protect myself so that I should not contract those communicable diseases like cholera, corona, syphilis, and gonorrhea.”* –ALWH, female, age 18, BDI-II 31 (severe)

This female ALWH explains how she received medical advice from her aunt, which is helpful in promoting her own well-being. Receiving advice from a trusted relative living with HIV can be particularly impactful in reducing stigma. They can share experiences navigating HIV care and promote accountability with ART adherence. In another example, a Periscope participant shares how important it is for family members (including those who do not have HIV) to encourage ART adherence:

*“She [sister] has to encourage me so that I can get motivated to look at taking medication as something normal.. we need support from them, to tell us to take medication, so we can be healthy.”* –Periscope participant, female, age 21

This participant reflects how her sister’s ART encouragement signals a larger acceptance of one’s HIV status. Feeling accepted in this way is integral in overcoming stigma, adhering to ARTs, and living a healthy life. On the contrary, discouragement of ARTs leads to detrimental downstream health effects, namely opportunisitic infections and HIV progression.

##### Psychosocial support

Despite often facing stigma, participants note that ALWH can find psychosocial support, primarily by talking to friends and family members. This quote illustrates some of the positive support an ALWH may receive:

*“Okay, the way I am I can be told unpleasant things because of my [HIV] status which affects me. So, this person is by my side enlightening me … “You should not be stressed very much, there are a lot of people who have HIV and they are living okay with their life. It’s not like they are suffering, their life is okay and stable. They are not stressed. If you are like those people, that is going to help you fulfill your dreams.” That’s some of the advice.”* –ALWH, male, age 17, BDI-II 13 (minimal)

This male ALWH describes how he needs someone to provide psychosocial support that challenges prevailing inaccurate HIV stereotypes. He specifically cites wanting future-oriented advice, inspiration to fufill one’s dreams, and dispelling myths that HIV is a death sentence. These words of encouragement can help ALWH resist downstream effects of HIV stigma, such as depression and anxiety.

### Health and social impacts

#### Morbidity

##### Poor mental health

Participants reported that HIV stigma is a major contributor to poor mental health, including symptoms of anxiety and depression. In the example that follows, the participant notes that his symptoms of depression escalated significantly after his HIV status was disclosed by his mother.

*“When I told my relatives and parents, they took it well. But some relatives couldn’t keep it a secret. When we had a disagreement, they would start talking about the condition [HIV]. This made the feelings [of depression] start escalating little by little, up to a point that they were overwhelming. This made me feel sad and it would take me a lot of time to come back to my normal state.”* – ALWH, male, age 18, BDI-II 21 (moderate)

As illustrated in this example, HIV status disclosure, as the trigger to the deterioration of one’s mental health, was a common narrative portrayed by ALWH.

##### Substance use

One participant spoke about using substances to cope with the psychological distress of living with a chronic, highly stigmatized disease such as HIV.

*“Of course, sometimes I do get stressed about it [my health] … We are worried about it because we are not okay, even though we look strong… Our condition exposes us to a great vulnerability needing comprehensive care, which in true sense, we can’t afford. So, because of that our health gets compromised. So, with that, and to fit in, you find yourself involved in drug and substance abuse which again destroys you… It is capable of destroying our lives.”* – ALWH, male, age 19, BDI-II 17 (mild)

In addition to using substances to relieve stress, this male ALWH notes that substance use is a way to fit in with peers and overcome the social isolation created by HIV. He recognizes the harmful effects of substance use on one’s health, which may exacerbate challenges in engaging in HIV care, as well as the physical health concerns associated with HIV.

##### Suicidality

Participants also discussed the close link between HIV stigma and suicidal ideation. This male ALWH describes how HIV status disclosure leads to HIV stigma, including mocking and internalized stigma, which results in severe anxiety. Furthermore, this participant believes that if his HIV status was to be disclosed, it could lead to his own suicide.

*“Someone disclosing your HIV status makes me anxious because it can spoil a lot of things… having stresses and maybe worrying… so you lose confidence. When people are laughing about their own things, you think they are laughing at you… So, it is difficult, and I worry that if it happens that way, then that’s the end of me. So, because of such a high level of anxiety, you can end up committing suicide.”* –ALWH, male, age 19, BDI-II 17 (mild)

In this example, the anxiety resulting from concerns over HIV status disclosure and HIV stigma are directly linked to suicidal ideation. A Periscope participant shared a similar sentiment, connecting HIV stigma and suicide for ALWH.

*“He can stop taking medication because the best option for him is to die, or maybe think of hanging himself, thinking that he is being stigmatized because he has HIV.”* – Periscope participant, female, age 22

In this manner, she notes that for an ALWH, HIV stigma can have such a negative impact on their self-worth and self-esteem that they might feel their only option is to take their own life.

#### Mortality

While not explicitly stated by participants, the aforementioned discussion of the adverse impact of stigma on access to and engagement in care as well as increased morbiditiy implies that individuals connect stigma with increased likelihood of mortality due to HIV. For example, in the quotes presented throughout this manuscript, many participants describe a belief that those who stop taking their ART will die from AIDS complications or suggest that the impact of stigma on mental health may cause premature death from suicide.

## Discussion

In this qualitative study, we used the HSD framework [24] to explore the stigmatization process faced by ALWH through interviews with ALWH with depressive symptoms, caregivers of ALWH, HIV providers, and staff and young participants of a previous depression counseling intervention. We found that HIV stigma adversely impacts HIV care engagement for ALWH in Malawi. Further, manifestations of HIV stigma, including gossip, insults and mocking, and physical and social distancing, can contribute to internalized stigma and poor mental health. Lastly, social support may be particularly important for withstanding stigma for ALWH.

This study confirms the known negative impact of stigma on HIV care engagement, particularly appointment attendance and adherence to ARTs. Participants cited decreased motivation to attend clinic appointments, due to fear of HIV status disclosure. This finding is consistent with prior studies in Malawi [16] and SSA [13–15]. Parents also desired privacy, even at the expense of withholding care for their children, which highlights the integral role that stigma and parents play in HIV care engagement for ALWH. ART nonadherence was driven by two processes: discouragement around lifelong medications and negative community attitudes towards ARTs. The requirement to take lifelong HIV medications created feelings of otherness for ALWH, leading to internalized stigma, and potentially depression, further driving decreased adherence to one’s daily ART regimen. Community members held negative perceptions of ARTs, as ARTs served as “marker” of HIV. ALWH described how they would discontinue ARTs, thus removing the “marker” of stigma, to fit in with their peers. Participants also cited a fear that ARTs can cause sudden death, a belief that reflects pervasive misconceptions around medication effects and HIV disease progression. ART nonadherence due to HIV stigma has been documented in SSA [16, 42, 43] and worldwide [17, 44, 45] and has serious implications for quality of life, viral suppression, morbidity, and mortality.

Manifestations of HIV stigma included gossip, insults and mocking, and physical and social distancing. In our study population, these manifestations were anticipated in all facets of an ALWH’s life, particularly following HIV status disclosure. Similarly, another Malawian study found that ALWH are subject to gossip and mockery and are excluded from playing with peers without HIV [16]. Further, other studies from across SSA have shown bullying of ALWH is common [14, 42, 43, 46, 47]. During the transition to adulthood, individuals begin identifying more with peers and less with parents, and belonging to a peer group is important for healthy psychological adjustment and academic achievement [4]. Gossip, mockery, physical distancing, and exclusion from social activities can thus cause severe psychological distress through feelings of alienation, disconnectedness, and poor self-image [4]. Such manifestations of stigma can, in turn, contribute to internalized stigma for ALWH, which is known to have a strong association of depression [48].

Our study highlighted that HIV stigma has major consequences for the mental health of ALWH. Depression, anxiety, and suicide risk were commonly cited outcomes of stigma, often occurring following HIV status disclosure, as was unhealthy substance use. Depression is often driven by internalized stigma, which is marked by feelings of shame and worthlessness, as seen in Malawi [16, 23, 49, 50], SSA [42, 43], and worldwide [51]. Additionally, many study participants closely linked HIV status disclosure and suicide risk. The increased risk of suicide amongst ALWH, and the link between HIV stigma and suicidality, has been documented in Malawi and other countries in the region [16, 52–55]. Some participants in our study also reported a preference for ART discontinuation with the intention of eventual death, as death would be preferable to indefinite ostracization. This method of “slow suicide” for people living with HIV has been documented in the region [42]. Our study participants also described unhealthy substance use as a coping mechanism to overcome isolation created by HIV stigma. While other studies have documented unhealthy substance use amongst ALWH [56–58], the role of stigma on substance use merits further investigation. The impact of HIV stigma on mental health is particularly worrisome as adolescents are already predisposed to the development of lifelong mental health disorders during this age [59].

ALWH identified social support as paramount in fostering resilience against stigma. Participants specifically cited the importance of friends and family supporting their HIV care, for example, by providing reminders to take ARTs. Encouragement to take ARTs can be interpreted as investment and caring for an ALWH’s life and even acceptance of one’s HIV status. ART encouragement may be important for supporting positive coping [17] and depression treatment [60]. ALWH also identified a need for friends and family to challenge inaccurate stereotypes, particularly that HIV is a death sentence, and inspire them to achieve their dreams. This supports other findings that suggest social support, particularly from family, may play an integral role in stigma-reduction efforts and mental health intervention for ALWH [61–64]. Notably, HIV status disclosure is a precursor for receiving social support, particularly from friends, and studies suggest that status disclosure can increase feelings of social support [65–67]. However, status disclosure can also undermine relationships, so it is necessary to advocate for healthy, safe, and selective disclosure for ALWH with the ultimate goal of fostering social support.

### Recommendations for future directions

Stigma-reduction interventions for adolescents in low- and middle- income countries is an underrepresented area of research [68]. Most stigma-reduction interventions for children and adolescents have been implemented in school-based settings [68]; however, to effectively tackle stigma, interventions are needed across multiple socioecological levels [9, 69]. To address public stigma, the most effective strategies are education and social contact between people with lived experience of stigma and the general public [68]. Additionally, addressing internalized stigma, either through individual or group-based counseling, represents an area for critical intervention, as internalized stigma is highly associated with poor mental health [48, 70]. Broadly, our findings support increased investment in comprehensive mental health services for ALWH, including stigma-reduction programming that is tailored to the unique developmental needs of adolescents. Implementation science should be leveraged to identify how we can best integrate evidence-based mental health interventions attuned to stigma into existing HIV care programming [71]. Stigma-reduction interventions are critical in supporting ALWH to improve their mental health, engage in HIV care, establish healthy habits as they transition into adulthood, and prevent adverse health outcomes.

### Limitations

There are some inherent limitations to this study. First, the participants were purposively sampled from peri-urban clinics in Lilongwe, limiting the generalizability of our findings to other regions of Malawi, particularly rural regions or other countries in SSA. Second, Periscope participants may face stigma due to multiple conditions, such as from HIV, teen pregnancy, and perinatal depression, etc., so their experiences may not be reflective of the general ALWH population. However, due to their recent experiences with HIV, depression, and engaging in mental health treatment at a young age, their insights are incredibly valuable. Third, this analysis draws from a larger formative study focused on adapting a counseling and peer support intervention for ALWH. As such, some interview questions inherently facilitated conversations focused on social support and HIV care engagement. Additionally, certain manifestations of stigma, such as affiliated stigma, were not explored in the interviews due to the nature of the formative work. Despite these limitations, this study provides valuable insights into the experience of stigma faced by ALWH and experiencing depressive symptoms in Malawi.

## Conclusion

This qualitative study uses the HSD Framework to systematically describe the stigmatization process faced by ALWH and comorbid depression in Malawi, expanding the small, yet growing, body of knowledge about how stigma is experienced in this vulnerable population. Identified drivers of HIV stigma include fear of HIV transmission, negative effects of ARTs, association with death, inaccurate knowledge, and negative attitudes towards ALWH. ALWH experience gossip, mockery, and social exclusion due to their HIV status. Notably, ALWH are at risk for negative mental health outcomes, including depression, anxiety, unhealthy substance use, and suicidality. These findings highlight the need to develop multi-level stigma reduction interventions that meet the mental health needs of ALWH and ultimately disrupt the adverse impact of stigma on mental health and HIV outcomes.

## Supplementary Information

Additional file 1: Interview Guides and Table S1. Interview Guides for Formative Phase of HEADS-UP Study and Table S1- Representative Interview Questions, specified by topic and participant type. This file includes four interview guides (IDI for ALWH, FGD for ALWH, IDI for Previous Periscope Participants and Implementors, FGD for Stakeholders) and one social support mapping session guide for ALWH. This file also contains a table that depicts pertinent interview questions from which the stigma data emerged that is presented in this manuscript, and shows how similar questions were asked to different participants.

Additional file 2. Codebook for HEADS-UP Formative Phase. This file includes the finalized codebook for the formative phase of the HEADS-UP study. It details parent, child, and grandchild codes specific to depression and HIV experiences.

Additional file 3. COREQ Checklist. COREQ (Consolidated criteria for Reporting Qualitative research) Checklist. This file includes a 32-item checklist that covers important considerations for qualitative research studies.

## Data Availability

Due to the discussion of sensitive topics, the datasets generated and/or analysed during the current study are not publicly available to protect study participant privacy. They may be available from the corresponding author on reasonable request via email at maria_faidas@med.unc.edu.

## Abbreviations

ALWH: Adolescents living with HIV
ART: Antiretroviral therapy
SSA: Sub-Saharan Africa
HSD: Health Stigma and Discrimination
FB: Friendship Bench
BDI-II: Beck-Depression Inventory-II
IDI: In-depth interview
FGD: Focus-group discussion
COREQ: COnsolidated criteria for REporting Qualitative research
MTCT: Mother-to-child transmission

## References

[1] World Health Organization. Adolescent mortality ranking-top 5 causes. 2024.

[2] UNICEF. Adolescent HIV treatment. 2023. https://data.unicef.org/topic/hivaids/adolescent-hiv-treatment/.

[3] Nachega JB, et al. Antiretroviral therapy adherence, virologic and immunologic outcomes in adolescents compared with adults in southern Africa. J Acquir Immune Defic Syndr. 2009;1999(51):65–71. 10.1097/QAI.0b013e318199072e PMC2674125 19282780

[4] Lerner RM, Steinberg L. Handbook of adolescent psychology. Hoboken: Wiley; 2009.

[5] University of Cape Town and University of Oxford. Mental health and antiretroviral treatment adherence among adolescents living with HIV: policy brief. 2021. https://www.unicef.org/esa/media/10231/file/Mental-Health-Treatment-Adhrence-Policy-Brief-2021.pdf.

[6] Kim MH, et al. High self-reported non-adherence to antiretroviral therapy amongst adolescents living with HIV in Malawi: barriers and associated factors. J Int AIDS Soc. 2017;20:21437. 28406275 10.7448/IAS.20.1.21437 PMC5515061

[7] Kamen C, et al. HIV-related stigma: implications for symptoms of anxiety and depression among Malawian women. Afr J AIDS Res AJAR. 2015;14:67–73. 25920985 10.2989/16085906.2015.1016987 PMC4416225

[8] Hatzenbuehler ML, Phelan JC, Link BG. Stigma as a fundamental cause of population health inequalities. Am J Public Health. 2013;103:813–21. 23488505 10.2105/AJPH.2012.301069 PMC3682466

[9] Nyblade L, Mingkwan P, Stockton MA. Stigma reduction: an essential ingredient to ending AIDS by 2030. Lancet HIV. 2021;8:e106–13. 33539757 10.1016/S2352-3018(20)30309-X

[10] Link BG, Phelan JC. Conceptualizing stigma. Annu Rev Sociol. 2001;27:363–85.

[11] Joint United Nations Programme on HIV/AIDS. Protocol for the identification of discrimination against people living with HIV. UNAIDS Geneva Switz; 2000. p. 4–9. https://data.unaids.org/publications/irc-pub01/jc295-protocol_en.pdf.

[12] Mahajan AP, et al. Stigma in the HIV/AIDS epidemic: a review of the literature and recommendations for the way forward. AIDS Lond Engl. 2008;22:S67–79. 10.1097/01.aids.0000327438.13291.62 PMC2835402 18641472

[13] Kimera E, Vindevogel S, Engelen AM, De Maeyer J, Reynaert D, Kintu MJ, et al. HIV-related stigma among youth living with HIV in Western Uganda. Qual Health Res. 2021;31(10):1937–50. 10.1177/10497323211012347. 33980098 10.1177/10497323211012347

[14] Pantelic M, Casale M, Cluver L, Toska E, Moshabela M. Multiple forms of discrimination and internalized stigma compromise retention in HIV care among adolescents: findings from a South African cohort. J Int AIDS Soc. 2020;23:e25488. 32438498 10.1002/jia2.25488 PMC7242009

[15] Embleton L, et al. Intersectional stigma and implementation of HIV prevention and treatment services for adolescents living with and at risk for HIV: opportunities for improvement in the HIV continuum in sub-Saharan Africa. AIDS Behav. 2023;27:162–84. 35907143 10.1007/s10461-022-03793-4 PMC10192191

[16] Kip EC, Udedi M, Kulisewa K, Go VF, Gaynes BN. Stigma and mental health challenges among adolescents living with HIV in selected adolescent-specific antiretroviral therapy clinics in Zomba District, Malawi. BMC Pediatr. 2022;22:253. 35524228 10.1186/s12887-022-03292-4 PMC9077887

[17] Katz IT, et al. Impact of HIV-related stigma on treatment adherence: systematic review and meta-synthesis. J Int AIDS Soc. 2013;16:18640. 24242258 10.7448/IAS.16.3.18640 PMC3833107

[18] Chambers LA, et al. Stigma, HIV and health: a qualitative synthesis. BMC Public Health. 2015;15:848. 26334626 10.1186/s12889-015-2197-0 PMC4557823

[19] Singleton L. Developmental differences and their clinical impact in adolescents. Br J Nurs Mark Allen Publ. 2007;16:140–3. 10.12968/bjon.2007.16.3.22965 17363878

[20] Tsai AC. Reliability and validity of depression assessment among persons with HIV in sub-Saharan Africa: systematic review and meta-analysis. J Acquir Immune Defic Syndr. 2014;66:503–11. 24853307 10.1097/QAI.0000000000000210 PMC4096047

[21] Gaynes BN, et al. Prevalence and predictors of major depression in HIV-infected patients on antiretroviral therapy in Bamenda, a semi-urban center in Cameroon. PLoS One. 2012;7:e41699. 22860006 10.1371/journal.pone.0041699 PMC3409230

[22] Lofgren S, Bond D, Nakasujja N, Boulware DR. Burden of depression in outpatient HIV-infected adults in sub-Saharan Africa; systematic review and meta-analysis. AIDS Behav. 2020;24:1752–64. 31720956 10.1007/s10461-019-02706-2 PMC7478178

[23] Kim MH, et al. Prevalence of depression and validation of the Beck Depression Inventory-II and the Children’s Depression Inventory-Short amongst HIV-positive adolescents in Malawi. J Int AIDS Soc. 2014;17:18965. 25085002 10.7448/IAS.17.1.18965 PMC4119288

[24] Stangl AL, et al. The Health Stigma and Discrimination Framework: a global, crosscutting framework to inform research, intervention development, and policy on health-related stigmas. BMC Med. 2019;17:31. 30764826 10.1186/s12916-019-1271-3 PMC6376797

[25] Dao TT, et al. Friendship Bench intervention to address depression and improve HIV care engagement among adolescents living with HIV in Malawi: study protocol for a pilot randomized controlled trial. medRxiv. 2024:2024.04.11.24305686. 10.1101/2024.04.11.24305686. 10.1371/journal.pone.0302666 PMC11922207 40106412

[26] Chibanda D, et al. Effect of a primary care-based psychological intervention on symptoms of common mental disorders in Zimbabwe: a randomized clinical trial. JAMA. 2016;316:2618–26. 28027368 10.1001/jama.2016.19102

[27] Ouansafi I, Chibanda D, Munetsi E, Simms V. Impact of Friendship Bench problem-solving therapy on adherence to ART in young people living with HIV in Zimbabwe: a qualitative study. PLoS One. 2021;16:e0250074. 33886619 10.1371/journal.pone.0250074 PMC8061927

[28] Bengtson AM, et al. An intervention to improve mental health and HIV care engagement among perinatal women in Malawi: a pilot randomized controlled trial. AIDS Behav. 2023:1–12. 10.1007/s10461-023-04070-8. 10.1007/s10461-023-04070-8 PMC10119837 37084104

[29] Akiba CF, et al. The Sub-Saharan Africa Regional Partnership (SHARP) for mental health capacity building: a program protocol for building implementation science and mental health research and policymaking capacity in Malawi and Tanzania. Int J Ment Health Syst. 2019;13:70. 31728158 10.1186/s13033-019-0327-2 PMC6842238

[30] Gaynes BN, et al. The Sub-Saharan Africa Regional Partnership (SHARP) for mental health capacity-building scale-up trial: study design and protocol. Psychiatr Serv Wash DC. 2021;72:812–21. 10.1176/appi.ps.202000003 PMC8187465 33291973

[31] Stockton MA, et al. A mixed-methods process evaluation: integrating depression treatment into HIV care in Malawi. Glob Health Sci Pract. 2021;9:611–25. 34593585 10.9745/GHSP-D-20-00607 PMC8514021

[32] Udedi M, et al. Integrating depression management into HIV primary care in central Malawi: the implementation of a pilot capacity building program. BMC Health Serv Res. 2018;18:593. 30064418 10.1186/s12913-018-3388-z PMC6069990

[33] Beck AT, Steer RA, Brown G. Beck depression inventory–II. 1996. 10.1037/t00742-000.

[34] Wheeldon J, Faubert J. Framing experience: concept maps, mind maps, and data collection in qualitative research. Int J Qual Methods. 2009;8:68–83.

[35] Herz A, Peters L, Truschkat I. How to do qualitative strukturale analyse? Die qualitative interpretation von Netzwerkkarten und erzählgenerierenden interviews. Forum Qual Sozialforschung Forum Qual Soc Res. 2015;16:8–16.

[36] Altissimo A. Combining egocentric network maps and narratives: an applied analysis of qualitative network map interviews. Sociol Res Online. 2016;21:152–64.

[37] Saldaña J. The coding manual for qualitative researchers. Sage; 2015.

[38] Gibbs G. Thematic coding and categorizing. In: Analyzing qualitative data. Sage; 2007. p. 38–56.

[39] Dedoose Version 9.0.17. Coud application for managing, analyzing, and presenting qualitative and mixed method research data. Sociocult Res Consult LLC Los Angel CA. 2021.

[40] Braun V, Clarke V. Thematic analysis. In: APA handbook of research methods in psychology, vol 2: research designs: quantitative, qualitative, neuropsychological, and biological. Washington, DC: American Psychological Association; 2012. p. 57–71. 10.1037/13620-004.

[41] Tong A, Sainsbury P, Craig J. Consolidated criteria for reporting qualitative research (COREQ): a 32-item checklist for interviews and focus groups. Int J Qual Health Care. 2007;19:349–57. 17872937 10.1093/intqhc/mzm042

[42] Willis N, Mavhu W, Wogrin C, Mutsinze A, Kagee A. Understanding the experience and manifestation of depression in adolescents living with HIV in Harare, Zimbabwe. PLoS One. 2018;13:e0190423. 29298326 10.1371/journal.pone.0190423 PMC5752002

[43] Dow DE, et al. Evaluating mental health difficulties and associated outcomes among HIV-positive adolescents in Tanzania. AIDS Care. 2016;28:825–33. 26837437 10.1080/09540121.2016.1139043 PMC4905805

[44] Rao D, Kekwaletswe TC, Hosek S, Martinez J, Rodriguez F. Stigma and social barriers to medication adherence with urban youth living with HIV. AIDS Care. 2007;19:28–33. 17129855 10.1080/09540120600652303

[45] Martinez J, et al. The impact of stigma on medication adherence among HIV-positive adolescent and young adult females and the moderating effects of coping and satisfaction with health care. AIDS Patient Care STDs. 2012;26:108–15. 22149767 10.1089/apc.2011.0178 PMC3266519

[46] Berner-Rodoreda A, Ngwira E, Alhassan Y, Chione B. “Deadly”, “fierce”, “shameful”: notions of antiretroviral therapy, stigma and masculinities intersecting men’s life-course in Blantyre, Malawi. BMC Public Health. 2021;21:2247. 34893060 10.1186/s12889-021-12314-2 PMC8665632

[47] Kimera E, et al. Experiences and effects of HIV-related stigma among youth living with HIV/AIDS in western Uganda: a photovoice study. PLoS One. 2020;15:e0232359. 32330206 10.1371/journal.pone.0232359 PMC7182188

[48] Ashaba S, et al. Community beliefs, HIV stigma, and depression among adolescents living with HIV in rural Uganda. Afr J AIDS Res AJAR. 2019;18:169–80. 31339461 10.2989/16085906.2019.1637912 PMC6774805

[49] Kim MH, et al. Factors associated with depression among adolescents living with HIV in Malawi. BMC Psychiatry. 2015;15:264. 26503291 10.1186/s12888-015-0649-9 PMC4624356

[50] Kumwenda MK, et al. Lived experiences of people living with HIV-a qualitative exploration on the manifestation, drivers, and effects of internalized HIV stigma within the Malawian context. PLoS One. 2023;18:e0284195. 37104484 10.1371/journal.pone.0284195 PMC10138249

[51] Mellins CA, Malee KM. Understanding the mental health of youth living with perinatal HIV infection: lessons learned and current challenges. J Int AIDS Soc. 2013;16:18593. 23782478 10.7448/IAS.16.1.18593 PMC3687078

[52] Ashaba S, et al. Internalized HIV stigma, bullying, major depressive disorder, and high-risk suicidality among HIV-positive adolescents in rural Uganda. Glob Ment Health. 2018;5:e22. 10.1017/gmh.2018.15 PMC6036650 29997894

[53] Zietz S, et al. Suicide behaviour among adolescents in a high HIV prevalence region of western Kenya: a mixed methods study. Glob Public Health. 2021;16:88–102. 32567992 10.1080/17441692.2020.1782964 PMC7752827

[54] Namuli JD, Nalugya JS, Bangirana P, Nakimuli-Mpungu E. Prevalence and factors associated with suicidal ideation among children and adolescents attending a pediatric HIV clinic in Uganda. Front Sociol. 2021;6:656739. 34212027 10.3389/fsoc.2021.656739 PMC8239397

[55] Brooks MJ, Burmen B, Olashore A, Gezmu A, Lowenthal E. Symptoms of depression, anxiety, and thoughts of suicide/self-injury in adolescents and young adults living with HIV in Botswana. Afr J AIDS Res. 2023;22:54–62. 37116112 10.2989/16085906.2023.2186252 PMC10787227

[56] Earnshaw VA, Kidman RC, Violari A. Stigma, depression, and substance use problems among perinatally HIV-infected youth in South Africa. AIDS Behav. 2018;22:3892–6. 29909588 10.1007/s10461-018-2201-7 PMC6635913

[57] Birungi C, et al. Substance use among HIV-infected adolescents in Uganda: rates and association with potential risks and outcome factors. AIDS Care. 2021;33:137–47. 32005076 10.1080/09540121.2020.1717419

[58] Morawej Z, et al. Prevalence and factors associated with substance use among HIV positive youth attending HIV care and treatment centers in Dodoma, Tanzania. AIDS Res Ther. 2022;19:65. 36566242 10.1186/s12981-022-00485-w PMC9789664

[59] Patel V, Flisher AJ, Hetrick S, McGorry P. Mental health of young people: a global public-health challenge. Lancet. 2007;369:1302–13. 17434406 10.1016/S0140-6736(07)60368-7

[60] Stockton M, et al. “Depression to me means…”: knowledge and attitudes towards depression among providers and ART patients in Malawi. Carol J Interdiscip Med. 2023;3:6–24.

[61] Casale M, Boyes M, Pantelic M, Toska E, Cluver L. Suicidal thoughts and behaviour among South African adolescents living with HIV: can social support buffer the impact of stigma? J Affect Disord. 2019;245:82–90. 30368074 10.1016/j.jad.2018.10.102

[62] Takada S, et al. The dynamic relationship between social support and HIV-related stigma in rural Uganda. Ann Behav Med. 2014;48:26–37. 24500077 10.1007/s12160-013-9576-5 PMC4104218

[63] Gariépy G, Honkaniemi H, Quesnel-Vallée A. Social support and protection from depression: systematic review of current findings in Western countries. Br J Psychiatry. 2016;209:284–93. 27445355 10.1192/bjp.bp.115.169094

[64] Jimu C, Govender K, Kanyemba R, Ngbesso M-JO. Experiences of intimate relationships, stigma, social support and treatment adherence among HIV-positive adolescents in Chiredzi District, Zimbabwe. Afr J AIDS Res. 2021;20:214–23. 34635020 10.2989/16085906.2021.1979059

[65] Lee S, Yamazaki M, Harris DR, Harper GW, Ellen J. Social support and HIV-status disclosure to friends and family: implications for HIV-positive youth. J Adolesc Health Off Publ Soc Adolesc Med. 2015;57:73–80. 10.1016/j.jadohealth.2015.03.002 PMC4478132 25940217

[66] Atuyambe LM, et al. HIV/AIDS status disclosure increases support, behavioural change and HIV prevention in the long term: a case for an urban clinic, Kampala, Uganda. BMC Health Serv Res. 2014;14:276. 24950958 10.1186/1472-6963-14-276 PMC4076501

[67] Smith R, Rossetto K, Peterson BL. A meta-analysis of disclosure of one’s HIV-positive status, stigma and social support. AIDS Care. 2008;20:1266–75. 18608080 10.1080/09540120801926977

[68] Hartog K, et al. Stigma reduction interventions for children and adolescents in low- and middle-income countries: systematic review of intervention strategies. Soc Sci Med 1982. 2020;246:112749. 10.1016/j.socscimed.2019.112749 PMC7313083 31978636

[69] Rao D, et al. A systematic review of multi-level stigma interventions: state of the science and future directions. BMC Med. 2019;17:41. 30770756 10.1186/s12916-018-1244-y PMC6377735

[70] Mpinga K, et al. Prevalence and correlates of internalized stigma among adults with HIV and major depressive disorder in rural Malawi. AIDS Care. 2023;35:1775–85. 37001058 10.1080/09540121.2023.2195609 PMC10544700

[71] Bhana A, Kreniske P, Pather A, Abas MA, Mellins CA. Interventions to address the mental health of adolescents and young adults living with or affected by HIV: state of the evidence. J Int AIDS Soc. 2021;24:e25713. 34164939 10.1002/jia2.25713 PMC8222850

