# Supplementary material for: Stigma and discrimination faced by adolescents living with HIV and experiencing depression in Malawi
Source: BMC Glob Public Health. 2024 Jul 1;2:39. doi: 10.1186/s44263-024-00072-3 (PMC11622908; doi:10.1186/s44263-024-00072-3)
Supplement: Supplementary file 1 — Additional file 1: Interview Guides and Table S1. Interview Guides for Formative Phase of HEADS-UP Study and Table S1- Representative Interview Questions, specified by topic and participant type. This file includes four interview guides (IDI for ALWH, FGD for ALWH, IDI for Previous Periscope Participants and Implementors, FGD for Stakeholders) and one social support mapping session guide for ALWH. This file also contains a table that depicts pertinent interview questions from which the stigma data emerged that is presented in this manuscript, and shows how similar questions were asked to different participants. [file 44263_2024_72_MOESM1_ESM.docx]

**Additional File 1: Interview Guides for Formative Phase of HEADS-UP Study**

**Guide 1- In-Depth Interview Guide for Adolescents Living with HIV**

Hello, I am working on behalf of researchers at UNC Project-Malawi. We are very interested in hearing your opinion on issues that impact young people’s ability to engage in HIV care and which strategies would be most useful to help them feel better and stay in HIV care.

**Overview**: The goal of this in-depth interview guide is to explore how adolescent’s living with HIV (ALWH) experience depression, how depression impacts engagement in HIV care, and what adolescents prefer for peer support.

1. Before this interview we asked you some questions about feeling sad and overwhelmed, being unable to get enjoyment from things, not having enough energy to do the things you normally do, or feeling hopeless and have a difficult time coping. These feelings are referred to as depression. Young people with depression may also experience changes in appetite, have trouble sleeping or concentrating, may feel irritated, or may consider physically harming themselves. You told us that you had experienced some of these feelings and symptoms over the past two weeks. I’d like to ask you some questions about them:
   1. Can you please tell me why you have been feeling this way?
   2. When did you start experiencing these feelings? How often have you experienced these feelings?
      1. Did you experience these feelings before or after you received an HIV diagnosis?
      2. [For those who were perinatally infected] Did you experience these feelings before or after you knew you had HIV?
   3. Have you sought help for these feelings? Why or why not? What kind of help or treatment have you received for these feelings? [Probe: If participant hasn’t sought clinical care, see if they know it exists]
   4. How have these feelings affected your ability to take care of yourself or spend time with your family, friends, or partners?
2. Now we would like to learn more about young people who have depression in your community.
   1. How do people in your community talk about young people who are depressed? How can community members tell they are depressed? What are some negative beliefs and stereotypes that persist about young people with depression in Malawi today?
   2. What do young people in your community with depression do to try to feel better?
   3. Can they talk with friends, neighbors, family, or partner about the feelings they are experiencing? Why or why not? Who do they usually talk to? What are these conversations like?
   4. How would healthcare providers or other services help them to feel better?
   5. What challenges or barriers do young people face to receiving treatment for depression, such as difficult in getting to the clinic, stigma or negative attitudes associated with depression, or lack of support from their friends and family?
   6. Do you think your family and friends would be supportive of you receiving treatment for depression? Why or why not?
3. We would like to offer a counseling program for young people with depression at this clinic and would like your help designing the program.
   1. What type of people should serve as counselors? What qualities should counselors have? [Probe: age, personality, etc.]
   2. Where should counseling sessions take place? [Probe: clinic, community, home, phone, etc.]. Why is that the best place? How frequently should sessions occur?
   3. When would be the best time for young people to receive counseling? If at the clinic, should they be organized to take place at the same time as your ART visit or on a special day? How would you feel if you someone you knew saw you attending counseling sessions?
   4. What are some concerns you or other young people might have about or challenges you might face attending counseling sessions?
4. Can you tell me about how having depression might impact taking medication, going to appointments, or taking care of their health ALWH?
   1. Outside of depression, what other barriers or challenges do young people face to attending HIV care and taking their medications?
   2. What can be done to help?
   3. Are your family and friends supportive of you receiving HIV care and treatment?
   4. How do people in your community treat young people living with HIV?
5. We are considering a program that combines counseling sessions for depression with peer support. Social support includes the many ways friends, family and others can help you. For example, they can give you emotional support when you feel down, they can give you advice or information when you are trying to solve a problem, they can be available when you want to spend time, or they could help you with a chore or give you money when you are in need.
   1. If you had to choose someone to support you with your HIV care, who would it be? Why?
   2. What qualities would they have? Is it important that the person also have HIV?
   3. How would they support you? What would you like them to do?
   4. [*ONLY IF PEER NOT MENTIONED ABOVE]* If you had to choose a **peer** to support you, who would it be? Why?
6. Now I’m going to explain different support peer strategies to you and get your thoughts on each.
   1. What do you think about having a peer accompany ALWH to the ART clinic or greet them and help them once they get to the ART clinic?
   2. What do you think about ALWH receiving text messages from a peer to remind them to attend their HIV visits?
   3. Do you think it would be better for peers to support ALWH individually or through a group with other ALWH?
   4. What do you think about ALWH having the opportunity to talk with a fellow peer who is also living with HIV about the problems they are experiencing?
   5. So far, we have talked about these strategies: peers accompanying you to the ART clinic, peers sending you reminder text messages, talking to peers individually, and talking to peers in a group. Can you tell me which of these strategies would be the most helpful? Why? Which would be the least helpful? Why? Are there any other strategies that would be helpful?

Do you have anything else you would like to tell me?

**Guide 2- Focus Group Guide for Adolescents Living with HIV**

Hello, I am working on behalf of researchers at UNC Project-Malawi. We are very interested in hearing your opinion on issues that impact young people’s ability to engage in HIV care and which strategies would be most useful to help them feel better and stay in HIV care.

**Overview:** The goal of this focus group guide is to explore how adolescent’s living with HIV (ALWH) experience depression, and which strategies might be useful to support engagement in HIV care among ALWH.

**Overview of Depression:**

1. Have you ever noticed someone your age who is feeling sad and lacks interest in their daily activities for an extended period? Or someone who is having trouble taking food, sleeping or concentrating, or engaging with their friends and family? They also might feel irritated or be considering physically harming themselves.
   1. How would you describe someone like this?

If no answer, further probe: What do you call it when someone is unhappy almost all of the time?

1. The words we use as researchers to describe people with these issues might be something like stress, anxiety, having worries or even depression. Have you heard those words before? [*If yes, ask a; if no, ask b*]
   1. If yes, how have you heard them used?
   2. If no, how would you describe when young people experience this type of sadness?
2. If a young person experiences depression, how do you think it may affect their engagement in HIV care?
   1. How might depression affect young people’s ability to take their HIV medications? What about attending their HIV appointments?
   2. What difficulties might they face?

**Counseling to Address Depression:**

1. We would like to offer a counseling program for young people with depression at this clinic and would like your help designing the program.
   1. What type of people should serve as counselors? What qualities should counselors have? [Probe: age, personality, etc.]
   2. Where should counseling sessions take place? [Probe, clinic, community, home, phone, etc. Probe on pros and cons)]. Why is that the best place? How frequently should sessions occur?
   3. When would be the best time for young people to receive counseling? If at the clinic, should they be organized to take place at the same time as ART visits or on a special day?
   4. What are some concerns you or other young people might have about attending counseling sessions? How would do you think young people would feel if they saw someone they knew while attending counseling sessions?

**Peer Support Strategies to Support Engagement in HIV Care:**

We are considering a program that combines counseling sessions with peer support. Social support includes the many ways friends, family and others can help you. For example, they can give you emotional support when you feel down, they can give you advice or information when you are trying to solve a problem, they can be available when you want to spend time, or they could help you with a chore or give you money when you are in need.

1. If you had to choose someone to support you with your HIV care, who would it be? Why?
   1. What qualities would they have? [Probe on age and other demographics and personality traits]
   2. How would they support you? What would you like them to do?
2. [*IF PEER NOT MENTIONED ABOVE]* If you had to choose a peer to support you, who would it be? Why?
   1. What qualities would they have?
   2. How would they support you? What would you like them to do?
3. Now, I’m going to explain different peer support strategies to you all and get your thoughts on each.
   1. What do you think about ALWH having the opportunity to talk with a peer who is also living with HIV about the problems they are experiencing?
   2. What do you think about having a peer accompany ALWH to the ART clinic or greet them and help them once they get to the ART clinic?
   3. What do you think about ALWH receiving text messages from a peer to remind them to attend their HIV visits?
   4. Do you think it would be better for peers to support ALWH individually or through a group with other ALWH?
   5. So far, we have talked about these strategies: peers accompanying you to the ART clinic, peers sending you reminder text messages, talking to peers individually, and talking to peers in a group. Can you tell me which of these strategies would be the most helpful? Why? Which would be the least helpful? Why? Are there any other strategies that would be helpful?

Do you have anything else you would like to tell me?

**Guide 3- In-Depth Interview Guide for Previous Periscope Participants and Implementers**

Hello, I am working on behalf of researchers at UNC Project-Malawi. We hope that your observations and experience from the Periscope study, which adapted a talk therapy/depression counseling to work better for pregnant depressed women, can help us understand how to adapt this same talk therapy for young people (age 13-19) who are living with HIV and have depression.

**Overview**: The goal of this in-depth interview guide is to understand the experience of previous participants and implementers of a counseling based mental health intervention.

1. Many young people who are living with HIV feel sad and have a difficult time coping. They may lack interest in their daily activities for an extended period and have trouble taking food, sleeping or concentrating, or engaging with their friends and family. They also might possibly be feeling irritated or considering physically harming themselves. These feelings are referred to as depression.
   1. How common do you think depression is your community or clinic?
   2. How important of a health issue do you think depression is for young people living with HIV?
   3. In your experience, how does depression affect young people’s health? How does it affect their ability to engage in HIV care and on ART?
2. You were previously involved with a counseling intervention for pregnant women living with HIV who were experiencing depression. Can you please tell me about your experience with the counseling intervention?
   1. What was your role in relation to Periscope – participant or counselor?
   2. From your perspective, what was the main point of the counseling sessions?
   3. [*For participants]* What benefits did you expect to receive from the counseling sessions?
      1. *[For counselors]* What benefits did you expect the counseling sessions to offer participants/patients?
   4. Did the counseling sessions meet your expectations?
   5. What worked well with the counseling sessions?
   6. What did not work well about the counseling sessions?
   7. How would you improve the counseling sessions?
   8. [*For participants]* What barriers were there to attending counseling sessions?
      1. [*For counselors] What barriers did you face or how difficult was it to implement the counseling sessions?*
3. We are planning to provide counseling sessions for young people (age 13-19) living with HIV who are experiencing depression. Could you tell me how we might need to change the counseling sessions you experienced/implemented to adapt them for young people?

*Prompts*

- 1. Who should provide the counseling? What qualities should the counselor have? (Probe on age, other demographics, personality, etc.)
  2. Where should the counseling be provided? [Probe: clinic, community, home, phone, etc.]. Why is that the best place? How frequently should sessions occur? When would be the best time for young people to receive counseling?
  3. What should be included in the counseling? [Probe: topics, skills, etc.].
  4. What additional support do young people with HIV need to help them attend care and take their medication?
  5. Are there any other special concerns for young people living with HIV we should take into account when adapting the counseling intervention?

1. We would like to help young people with HIV, who experience depression, stay engaged in HIV care. We are considering a program that combines counseling sessions with social support. Social support includes the many ways friends, family and others can help us. For example, they can give you emotional support when you feel down, they can give you advice or information when you are trying to solve a problem, they can be available when you want to spend time, or they could help you with a chore or give you money when you are in need.
   1. If you had to choose someone to support young people with their HIV care, who would it be? (Probe options: peers, someone their age living with HIV, family, an older person, clinician, HTC counselor, etc.) Why?
   2. What qualities would they have? (Probe on age, other demographics, personality, etc.) Is it important that the person also have HIV?
   3. How would they support them? What would you like them to do? What advice would they give them?
2. Now I’m going to explain different support strategies to you and get your thoughts on each.
   1. What do you think about ALWH having the opportunity to talk with a fellow peer who is also living with HIV about the problems they are experiencing?
   2. What do you think about having a peer accompany ALWH to the ART clinic or greet them and help them once they get to the ART clinic?
   3. What do you think about ALWH receiving text messages from a peer to remind them to attend their HIV visits?
   4. Do you think it would be better for peers to support ALWH individually or through a group with other ALWH?
   5. So far, we have talked about these strategies: peers accompanying ALWH to the ART clinic, peers sending ALWH reminder text messages, ALWH talking to peers individually, and ALWH talking to peers in a group. Can you tell me which of these strategies would be the most helpful? Why? Which would be the least helpful? Why? Are there any other strategies that would be helpful?

Do you have anything else you would like to tell me?

**Guide 4- Focus Group Guide for Stakeholders (Providers and Caregivers)**

*Providers and Caregivers were interviewed in separate FGDs*

Hello, I am working on behalf of researchers at UNC Project-Malawi. We are very interested in hearing your opinion on issues that impact young people’s ability to engage in HIV care while experiencing depression, and which strategies would be most useful to help them stay in HIV care.

**Overview:** The goal of this focus group guide is to explore which strategies might be useful to support engagement in HIV care among adolescents living with HIV who are experiencing depression amongst parents, providers, and government health officials.

**Overview of Depression and HIV Care Engagement:**

1. Many young people who are living with HIV feel sad and have a difficult time coping. They may lack interest in their daily activities for an extended period and have trouble taking food, sleeping or concentrating, or engaging with their friends and family. They also might possibly be feeling irritated or considering physically harming themselves. These feelings are referred to as depression.
   1. How common do you think depression is in your community or clinic?
   2. How important of a health issue do you think depression is for young people living with HIV?
   3. In your experience, how does depression affect the health of a young person living with HIV? How does it affect their ability to take their medications or attend appointments? What other difficulties might they face?

**Counseling to Address Depression:**

1. In some clinics, providers offer counseling to help young people with depression.
2. In your experience, who do young people talk to about their problems at clinics? Why do you think young people are comfortable talking to them about their problems? What specific qualities do they have? How old are they? What is their role at the clinic?
3. [If No Conversation] Why do you think young don’t talk to anyone at the clinic about their problems?
4. For some young people with depression, talking with someone can help them to feel better. We would like to offer a counseling program for young people here in Lilongwe and would like your help designing the program.
   1. What type of people should serve as counselors? What qualities should counselors have? [Probe: age, personality, etc.]
   2. Where should counseling sessions take place? [Probe, clinic, community, home, phone, etc. Probe on pros and cons)]. Why is that the best place? How frequently should sessions occur?
   3. When would be the best time for young people to receive counseling? If at the clinic, should they be organized to take place at the same time as ART visits or on a special day?
   4. How do you think parents would feel about their children attending counseling services? Why do think that? What would make it easier for parents to support their children attending counseling services?
   5. What barriers are there to providing counseling services to young people? Probe: Government or policy barriers (resources, support, etc.), community barriers (cost, transport, stigma), or clinic barriers (time, staffing, privacy, importance)?

**Peer Support Strategies to Support Engagement in HIV Care:**

1. We are considering a program that combines counseling sessions with peer support. Social support includes the many ways friends, family and others can help an individual. For example, they can give someone emotional support when they feel down, they can give someone advice or information when they are trying to solve a problem, they can be available when someone wants to spend time, or they could help someone with a chore or give them money when they are in need.
   1. If you had to choose someone to support young people with their HIV care, who would it be? (Probe options: peers, someone their age living with HIV, family, an older person, clinician, HTC counselor, etc.) Why?
   2. What qualities would they have? (Probe on age, other demographics, personality, etc.) Is it important that the person also have HIV?
   3. How would they support them? What would you like them to do? What advice would they give them?
2. Now I’m going to explain different support strategies to you and get your thoughts on each.
3. What do you think about ALWH having the opportunity to talk with a peer who is also living with HIV about the problems they are experiencing?
4. What do you think about having a peer accompany ALWH to the ART clinic or greet them and help them once they get to the ART clinic?
5. What do you think about ALWH receiving text messages from a peer to remind them to attend their HIV visits?
6. Do you think it would be better for peers to support ALWH individually or through a group with other ALWH?
7. So far, we have talked about these strategies: peers accompanying ALWH to the ART clinic, peers sending ALWH reminder text messages, ALWH talking to peers individually, and ALWH talking to peers in a group. Can you tell me which of these strategies would be the most helpful? Why? Which would be the least helpful? Why? Are there any other strategies that would be helpful?

Do you have anything else you would like to tell me?

**Guide 5- Social Support Mapping Interview Guide for Adolescents Living with HIV**

Hello, I am working on behalf of researchers at UNC Project-Malawi. Social support is the many ways through which people assist each other. For example, people can give you emotional support when you feel down, they can give you advice or information when you are trying to solve a problem, they can be available when you want to spend time, or they could help you with a chore or give you money when you are in need. So today we are going to map your social support network and see how people may help you.

**Overview**: The goal of this in-depth interview guide is to explore ALWH’s social support networks in relation to depression and engagement in HIV care.

**SECTION 1: HIV CARE**

Let’s start by thinking about who might help you when you with your HIV care such as taking medication, going to appointments, or other things.

1. What is this person’s name?
   1. Probe: Who is this person? – *family, friend, neighbor, schoolmate, etc.*
   2. Probe: What are they like? – *personality, age, (if appropriate, HIV status), etc.*
   3. Probe: Where does this person live? – *same house, neighborhood, city or far?*
2. How close are you to this person - very close, somewhat close, or not close? Okay now let’s write their name on corresponding circle.
   1. Probe: Why do you feel very/somewhat/not close to this person?
   2. Probe: What sort of things do you do together? What sort of things do you talk about?
   3. Probe: How often do you see or talk to each other?
3. What kind of impact does this person have on your life? How much of an impact do they have on your life (small, medium, big)? Okay, now let’s write the corresponding symbol next to their name.
   1. Probe: Why?
   2. Probe: How does this person make you feel? What do they do that makes you feel this way?
4. What kind of support does this person give you? Let’s write the corresponding symbols next to their name.
   1. Probe: Can you tell me about that? Can you give me some examples?
   2. Probe: Do they give you any other types of support? (Allow participant to discuss multiple types of support from same person)

- Empathy
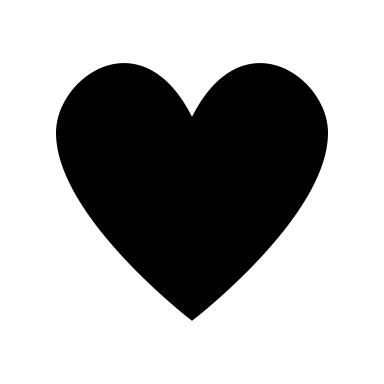

  - Emotional support is showing empathy, compassion, and genuine concern. This person listens to you and understands when you tell them about how you feel.
- Advice
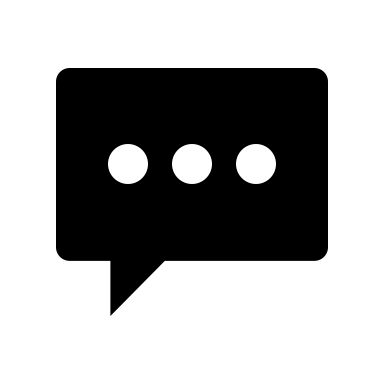

  - Advice or informational support is suggestions or information that you can use to work on problems. You would ask this person for advice and trust their judgment.
    - Probe: If participant says advice, probe on type of advice or advice content)
- Social
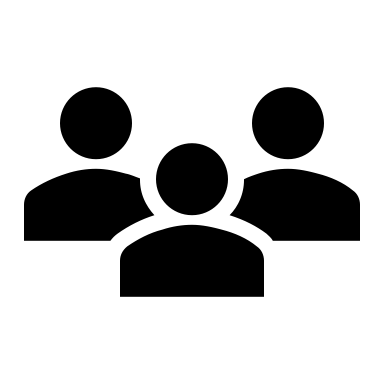

  - Social support is company that helps you to feel good about yourself. This is a person you like to hang out with.
- Practical
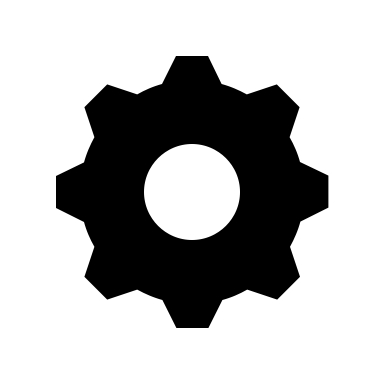

  - Practical support is help that makes your life more manageable. You can ask this person for things like money, a ride, or help taking care of your children.

1. Is there anything else you would like to say about this person? Okay, now let’s write that next to their name.

Now, who is another person who might help you with your HIV care? [Repeat until 3 people have been named]

**SECTION 2: DEPRESSION**

We’ve finished talking about people who might help you engage in HIV care. Now, let’s focus on who you think might help you when you are **feeling sad or depressed.** When we say depression, we mean when someone is feeling sad and lacks interest in their daily activities for an extended period. Young people with depression may also experience changes in appetite, have trouble sleeping or concentrating, may feel irritated, or may consider physically harming themselves

1. What is this person’s name? *[Note to interviewer, it’s okay if it’s one of the same people listed before, but still try to ask for examples of the different type of support]*
   1. Probe: Who is this person? – *family, friend, neighbor, schoolmate, etc.*
   2. Probe: What are they like? – *personality, age, (if appropriate, HIV status or mental health experience), etc.*
   3. Probe: Where does this person live? – *same house, neighborhood, city or far?*
2. How close are you to this person? Okay now let’s write their name on corresponding circle.
   1. Probe: Why do you feel very/somewhat/not close to this person?
   2. Probe: What sort of things do you do together? What sort of things do you talk about?
   3. Probe: How often do you see or talk to each other?
3. What kind of impact does this person have on your life? How much of an impact do they have on your life (small, medium, big)? Okay, now let’s write the corresponding symbol next to their name.
   1. Probe: Why?
   2. Probe: How does this person make you feel? What do they do that makes you feel this way?
4. What kind of support does this person give you? Let’s write the corresponding symbols next to their name.
   1. Probe: Can you tell me about that? Can you give me some examples?
   2. Probe: Do they give you any other types of support? (Allow participant to discuss multiple types of support from same person)?
   - Empathy
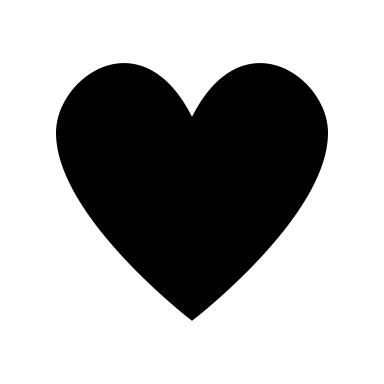

     - Emotional support is showing empathy, compassion, and genuine concern. This person listens to you and understands when you tell them about how you feel.
   - Advice
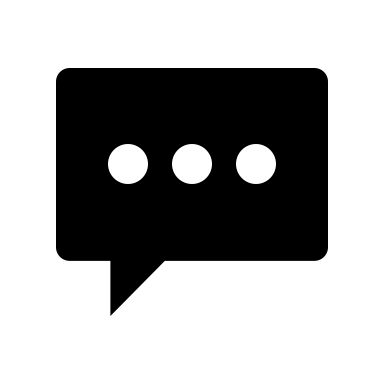

     - Advice or informational support is suggestions or information that you can use to work on problems. You would ask this person for advice and trust their judgment.
       - Probe: If participant says advice, probe on type of advice or advice content
   - Social
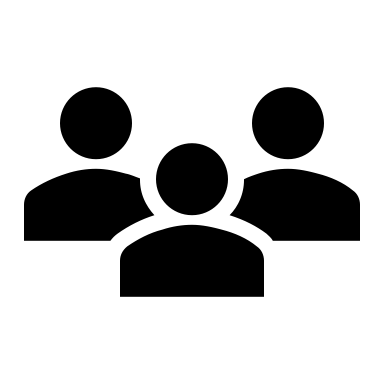

     - Social support is company that helps you to feel good about yourself. This is a person you like to hang out with.
   - Practical
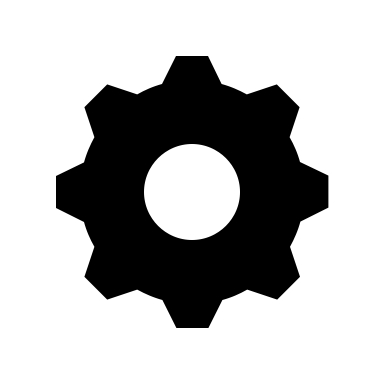

     - Practical support is help that makes your life more manageable. You can ask this person for things like money, a ride, or help taking care of your children.
       - Probe: If participant says advice, probe on type of advice or advice content
5. Is there anything else you would like to say about this person? [*Probe:* *encourage the participant to describe the person’s characteristics]* Okay, now let’s write that next to their name.

Now, who is another person who might help you when you are feeling sad or depressed? *[Repeat until 3 people have been named]*

Do you have anything else you would like to tell me?

**HIV Social Support Network Map**

| **Domain** | **Category** | **Symbol** | **Definition** |
| --- | --- | --- | --- |
| Closeness | Very close |  |  |
|  | Somewhat close |  |  |
|  | Not close |  |  |
| Impact | Small | + |  |
|  | Medium | ++ |  |
|  | Large | +++ |  |
| Support Type | Empathy | 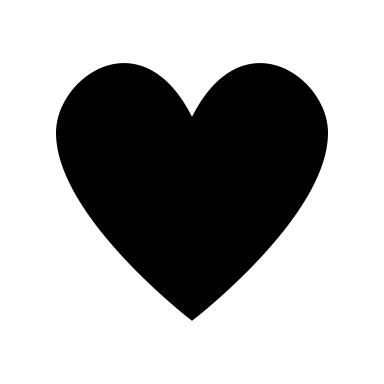 | Showing empathy, compassion, and genuine concern. This person listens to you and understands when you tell them about how you feel. |
|  | Advice | 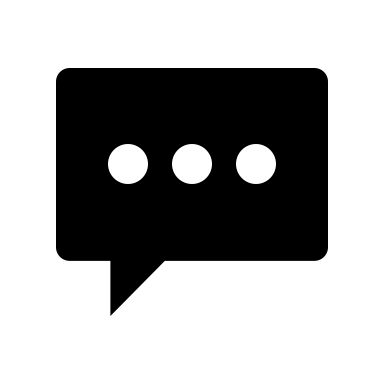 | Suggestions or information that you can use to work on problems. You would ask this person for advice and trust their judgment. |
|  | Social | 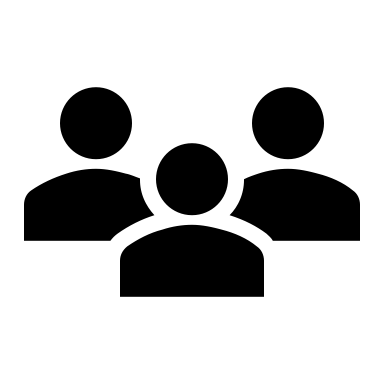 | Company that helps you to feel good about yourself. This is a person you like to hang out with. |
|  | Practical | 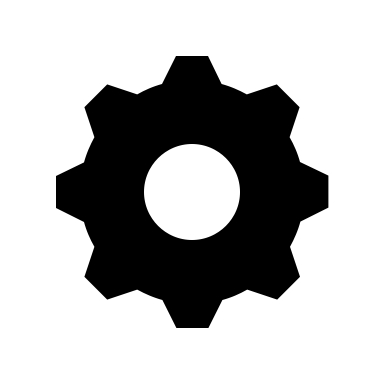 | Help that makes your life more manageable. You can ask this person for things like money, a ride, or help taking care of your children |


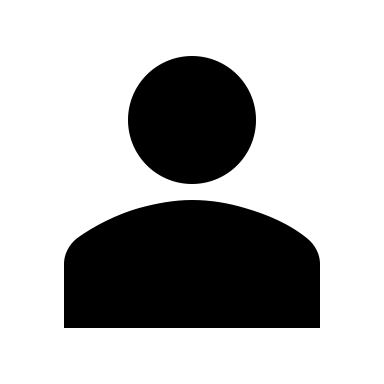


**ME**

**Depression Social Support Network Map**

| **Domain** | **Category** | **Symbol** | **Definition** |
| --- | --- | --- | --- |
| Closeness | Very close |  |  |
|  | Somewhat close |  |  |
|  | Not close |  |  |
| Impact | Small | + |  |
|  | Medium | ++ |  |
|  | Large | +++ |  |
| Support Type | Empathy | 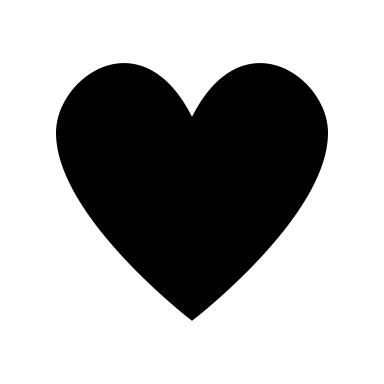 | Showing empathy, compassion, and genuine concern. This person listens to you and understands when you tell them about how you feel. |
|  | Advice | 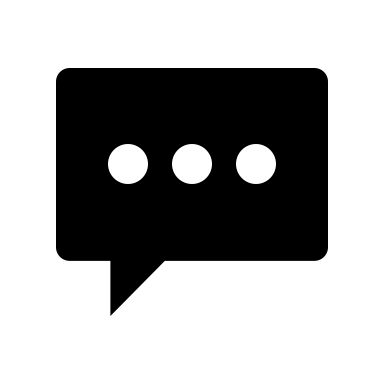 | Suggestions or information that you can use to work on problems. You would ask this person for advice and trust their judgment. |
|  | Social | 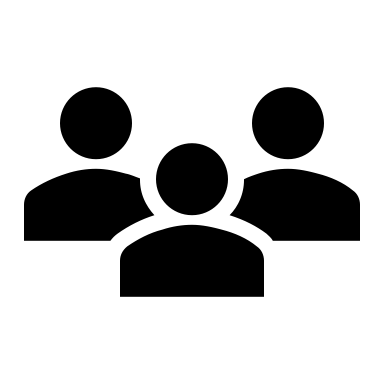 | Company that helps you to feel good about yourself. This is a person you like to hang out with. |
|  | Practical | 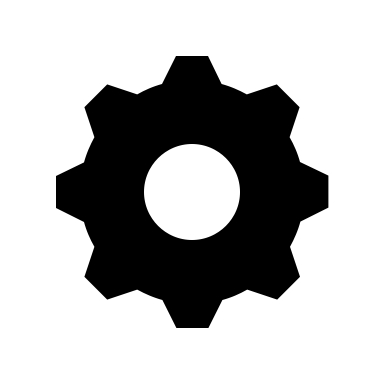 | Help that makes your life more manageable. You can ask this person for things like money, a ride, or help taking care of your children |


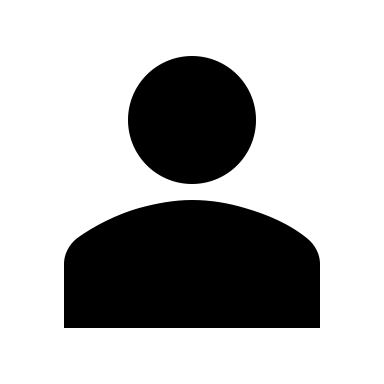


**ME**

**Table S1: Representative Interview Questions, specified by topic and participant type**

| **Topic** | **Participant Type** | | |
| --- | --- | --- | --- |
|  | **Adolescents living with HIV** | **Participants and Staff**  **in the Periscope Intervention** | **Stakeholders**  **(Caregivers, Providers)** |
| **Depression** | - Can you please tell me why you have been feeling depressed? - When did you start experiencing these feelings? How often have you experienced these feelings? - Have you sought help for these feelings? Why or why not? What kind of help or treatment have you received for these feelings? - How have these feelings affected your ability to take care of yourself or spend time with your family, friends, or partners? - How do people in your community talk about young people who are depressed? What are some negative beliefs and stereotypes that persist about young people with depression in Malawi today? | - How common do you think depression is your community or clinic? - How important of a health issue do you think depression is for young people living with HIV? - In your experience, how does depression affect young people’s health? | - How common do you think depression is your community or clinic? - How important of a health issue do you think depression is for young people living with HIV? - In your experience, how does depression affect the health of a young person living with HIV? |
| **Mental Health Care** | - What do young people in your community with depression do to try to feel better? - How would healthcare providers or other services help them to feel better? - What challenges or barriers do young people face to receiving treatment for depression, such as difficult in getting to the clinic, stigma or negative attitudes associated with depression, or lack of support from their friends and family? - Do you think your family and friends would be supportive of you receiving treatment for depression? Why or why not? | - Could you tell me how we might need to change the counseling sessions you experienced/implemented to adapt them for young people? - What barriers were there to attending counseling sessions? [for participants] - What barriers did you face or how difficult was it to implement the counseling sessions? [for staff] - Are there any other special concerns for young people living with HIV we should take into account when adapting the counseling intervention? | - Who do young people talk to about their problems at clinics? - What specific qualities do they have? - How do you think parents would feel about their children attending counseling services? What would make it easier for parents to support their children attending counseling services? - What barriers are there to providing counseling services to young people? Probe: Government or policy barriers (resources, support, etc.), community barriers (cost, transport, stigma), or clinic barriers (time, staffing, privacy, importance)? |
| **HIV Care and Support** | - Outside of depression, what other barriers or challenges do young people face to attending HIV care and taking their medications? - What can be done to help? - How do people in your community treat young people living with HIV? - If you had to choose someone to support you with your HIV care, who would it be? Why? - What kind of support would this person give you? | - How does depression affect their ability to engage in HIV care and on ART? - What additional support do young people with HIV need to help them attend care and take their medication? - If you had to choose someone to support young people with their HIV care, who would it be? (Probe options: peers, someone their age living with HIV, family, an older person, clinician, HTC counselor, etc.) Why? - How would they support them? What would you like them to do? What advice would they give them? | - How does depression affect their ability to take their medications or attend appointments? What other difficulties might they face? - If you had to choose someone to support young people with their HIV care, who would it be? (Probe options: peers, someone their age living with HIV, family, an older person, clinician, HTC counselor, etc.) Why? - How would they support them? What would you like them to do? What advice would they give them? |
