# Supplementary material for: Stigma and discrimination faced by adolescents living with HIV and experiencing depression in Malawi
Source: BMC Glob Public Health. 2024 Jul 1;2:39. doi: 10.1186/s44263-024-00072-3 (PMC11622908; doi:10.1186/s44263-024-00072-3)
Supplement: Supplementary file 3 — Additional file 3. COREQ Checklist. COREQ (Consolidated criteria for Reporting Qualitative research) Checklist. This file includes a 32-item checklist that covers important considerations for qualitative research studies. [file 44263_2024_72_MOESM3_ESM.docx]

**Additional File 3- COREQ (Consolidated criteria for Reporting Qualitative research) Checklist**

| **Domain** | **Item Number** | **Comment** | **Reported on Page Number or N/A** |
| --- | --- | --- | --- |
| **Domain 1: Research Team and Flexibility** | | | |
| *Personal Characteristics* | | | |
| Interviewer/Facilitator | 1 | Which author/s conducted the interview or focus group?  The authors did not conduct the interviews as they are not native Chichewa speakers. The research assistants, JN and MM, conducted the interviews and focus groups, who are included in the acknowledgements. | 9, 35 |
| Credentials | 2 | What were the researcher’s credentials? E.g. PhD, MD  MF- BA  MAS- PhD  SMM- BA  GS- BS  HH- BA  JD- MPH  TP- B.Ed, M.Sp.Ed  LR- MD  JK- BA  HM- MPH  KK- MBBS, MMed  NLB- PhD  BWP- PhD  BNG- MD, MPH | N/A |
| Occupation | 3 | What was their occupation at the time of the study?  MF is a medical student and research fellow. MAS is an assistant professor of psychiatry. SMM is a clinical research coordinator. GS is a medical student and former research intern. HH is a doctoral student. JD is a former graduate student. TP is a trainings and study coordinator. LR recently graduated from medical school. JK is a research intern. HM is a project manager. KK is a psychiatrist and head of psychiatry department. NLB is a research scientist. BWP is a professor of epidemiology. BNG is a psychiatrist and professor of psychiatry and epidemiology. | N/A |
| Gender | 4 | Was the researcher male or female?  There were 7 female and 7 male researchers. | N/A |
| Experience and Training | 5 | What experience or training did the researcher have?  MF has prior experience with qualitative research and is currently a research fellow in a global health training program. MAS has extensive experience in qualitative and quantitative research focusing on stigma and mental health. MAS and BNG mentored MF in data analysis and manuscript writing. SMM has been working as a research coordinator on mental health projects for over 10 years. HH is a former research analyst with an interest in mental health. JD is a graduate research assistant with experience in qualitative analysis. TP has 10 years of experience in qualitative research methods. LR has conducted research throughout medical school focusing on pediatric populations. JK is a mental health research intern. HM has prior experience as a research assistant. KK is a psychiatrist and mental health researcher. NLB is a research scientist with a background in health behavior. BWP and BNG have been conducting mental health research in Malawi for numerous years, combining their epidemiology and psychiatry backgrounds. | N/A |
| *Relationship with Participants* | | | |
| Relationship established | 6 | Was a relationship established prior to study commencement?  Interviewers (JN and MM) did not have any prior relationship with the participants to ensure the data collection was unbiased. | N/A |
| Participant knowledge of the interviewer | 7 | What did the participants know about the researcher? e.g. personal  goals, reasons for doing the research  Participants were introduced to the interviewers at the start of each IDI or FGD. During the informed consent process, participants were informed about the purpose of the study and data needed. All participants signed written informed consent. | 35 |
| *Interviewer characteristics* | 8 | What characteristics were reported about the interviewer/facilitator?  e.g. Bias, assumptions, reasons and interests in the research topic  Interviewer characteristics were not reported to participants to mitigate any potential bias. | N/A |
| ***Domain 2: Study Design*** | | | |
| *Theoretical framework* | | | |
| Methodological orientation and Theory | 9 | What methodological orientation was stated to underpin the study? e.g.  grounded theory, discourse analysis, ethnography, phenomenology,  content analysis  We used applied thematic analysis based on Braun et al. (2012). | 10 |
| Participant selection | | | |
| Sampling | 10 | How were participants selected? e.g. purposive, convenience,  consecutive, snowball  Participants were selected using purposive sampling. | 7 |
| Method of approach | 11 | How were participants approached? e.g. face-to-face, telephone, mail,  Email  ALWH were approached in-person during the depression screening process conducted during Teen Clubs. All other participants were contacted via phone. | 7- 8 |
| Sample Size | 12 | How many participants were in the study?  There were 42 total participants in this study. | 11 |
| Non-participation | 13 | How many people refused to participate or dropped out? Reasons?  No one refused to participate or dropped out. | 8 |
| Setting | | | |
| Setting of data collection | 14 | Where was the data collected? e.g. home, clinic, workplace  All IDIs and FGDs were conducted in private clinic rooms. | 9 |
| Presence of nonparticipants | 15 | Was anyone else present besides the participants and researchers?  Only interviewers and participants were present for IDIs and FGDs. | 9 |
| Description of sample | 16 | What are the important characteristics of the sample? e.g. demographic  data, date  All study sample characteristics are reported in the Results and Table 2. | 11-12 |
| *Data Collection* | | | |
| Interview guide | 17 | Were questions, prompts, guides provided by the authors? Was it pilot  tested?  The interview guides were developed based on prior Friendship Bench studies and present study objectives. The guides were reviewed for linguistic and cultural appropriateness. Interview guides were piloted prior to use. | 8 |
| Repeat interviews | 18 | Were repeat interviews carried out? If yes, how many?  No repeat interviews were conducted. | N/A |
| Audio/visual recording | 19 | Did the research use audio or visual recording to collect the data?  All interviews were audio recorded and saved on a secure platform. | 9 |
| Field notes | 20 | Were field notes made during and/or after the interview or focus group?  For each IDI and FGD, interviewers could note reflections and challenges on a cover sheet. These notes are saved in a locked cabinet. No issues were reported. | N/A |
| Duration | 21 | What was the duration of the interviews or focus group?  All data collection activities ranged from 1-1.5 hours. | 9 |
| Data saturation | 22 | Was data saturation discussed?  Data saturation was considered in the design of the target sample size and data saturation was reached. | N/A |
| Transcripts returned | 23 | Were transcripts returned to participants for comment and/or correction?  No transcripts were returned to participants. | N/A |
| **Domain 3: Analysis and Findings** | | | |
| *Data Analysis* | | | |
| Number of data coders | 24 | How many data coders coded the data?  There were 6 coders. | 10 |
| Description of the coding tree | 25 | Did authors provide a description of the coding tree?  Parent and child codes were created deductively to capture important topics in interview guides and inductively to capture emerging themes. | 10 |
| Derivation of themes | 26 | Were themes identified in advance or derived from the data?  Themes were identified both in advance and derived from the data, which is described in the codebook development. | 10 |
| Software | 27 | What software, if applicable, was used to manage the data?  Dedoose was used to manage the data. | 10 |
| Participant checking | 28 | Did participants provide feedback on the findings?  No participant checking was conducted. | N/A |
| Reporting | | | |
| Quotations presented | 29 | Were participant quotations presented to illustrate the themes/findings?  Was each quotation identified? e.g. participant number  Representative quotes were used to illustrate key themes and findings. Participants were kept anonymous and quotes were identified by participant type and basic demographics like age and gender. | 13-28 |
| Data and findings consistent | 30 | Was there consistency between the data presented and the findings?  There is consistency between data presented and the findings. All findings were reviewed by content experts. | N/A |
| Clarity of major themes | 31 | Were major themes clearly presented in the findings?  Yes, major themes are clearly identified. | N/A |
| Clarity of minor themes | 32 | Is there a description of diverse cases or discussion of minor themes?  Yes, minor themes are clearly identified. | N/A |

Developed from: Tong A, Sainsbury P, Craig J. Consolidated criteria for reporting qualitative research (COREQ): a 32-item checklist for interviews and focus groups. International Journal for Quality in Health Care. 2007. Volume 19, Number 6: pp.
